# Association of Myasthenia Gravis With Autoimmune Thyroid Disease: A Bidirectional Mendelian Randomization Study

**DOI:** 10.1002/brb3.70235

**Published:** 2025-01-19

**Authors:** Yao Wang, Ke Wang, Jing Lu, Peng Xu, Dongmei Zhang, Xinzhi Chen, Jian Wang

**Affiliations:** ^1^ College of Traditional Chinese Medicine Changchun University of Chinese Medicine Changchun China; ^2^ Research Center of Traditional Chinese Medicine The Affiliated Hospital to Changchun University of Chinese Medicine Changchun China; ^3^ Department of Neurology The Affiliated Hospital to Changchun University of Chinese Medicine Changchun China; ^4^ Scientific Research Office The Affiliated Hospital to Changchun University of Chinese Medicine Changchun China; ^5^ Department of Neurology The First Clinical Hospital Research Institute of Jilin Academy of ChineseMedicine Changchun China

**Keywords:** autoimmune thyroid disease | causation | genetics | Mendelian randomness | myasthenia gravis

## Abstract

**Background and Purpose:**

Observational studies have indicated a high occurrence of coexistence between myasthenia gravis (MG) and autoimmune thyroid disease (AITD) in clinical settings, but the causal relationship between the two conditions remains ambiguous. Therefore, this study endeavors to investigate the causal links between MG, along with its subgroups, and AITD through a Mendelian randomization (MR) approach.

**Methods:**

Genetic instrumental variables associated with MG and AITD were selected from three major publicly available GWAS databases for MR analysis. The primary method for evaluating causal effects was the inverse variance weighted (IVW) method. Supplementary methods included MR‐Egger regression and weighted median. The reliability and stability of the results were ensured through tests for heterogeneity, assessment of pleiotropy, and sensitivity analysis using the leave‐one‐out approach.

**Results:**

The investigation revealed reciprocal causal associations between MG and both Graves’ disease and autoimmune hypothyroidism. Genetic predisposition to MG was linked to an increased likelihood of developing Hashimoto's thyroiditis (OR = 1.242(1.073–1.437, *P* = 0.0036)), and early‐onset MG also exhibited an association with an elevated risk of HT (OR = 1.157(1.073‐1.246), *P* = 1.269×10^−4^). No statistically significant relationships were found for the other conditions.

**Conclusion:**

This extensive MR analysis provides evidence suggesting a potential association between MG and AITD, particularly with Graves' disease and Hashimoto's thyroiditis. Consequently, proactive treatment strategies targeting either MG or autoimmune thyroid disorders may help mitigate the risk of comorbidities in affected patients.

## Graphical abstract

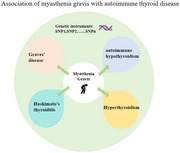

## 1 Introduction

Myasthenia gravis (MG) is a chronic autoimmune disorder of the nervous system characterized by recurring episodes of weakness and fatigue, primarily affecting skeletal muscles throughout the body, including the extraocular muscles (Hong et al. 2017). The prevalence of MG is approximately 150‐250 cases per million, with an annual incidence rate of around 4‐10 cases per million. Notably, the incidence of MG is currently on the rise (Heldal et al. 2009, Carr et al. 2010). The incidence of MG also exhibits a bimodal distribution with respect to gender and age: in women, the peak incidence occurs around the age of 30, while in men, it is delayed until after age 50 (Vinciguerra et al. 2023). MG is recognized as an antibody‐mediated neuromuscular disorder, primarily targeting acetylcholine receptors (AChR) and, to a lesser extent, muscle‐specific tyrosine kinase (MuSK). However, in a small subset of patients with ‘serum‐negative MG,’ no detectable autoantibodies are present, and the underlying mechanism remains unclear (Vinciguerra et al. 2023). MG often coexists with various other autoimmune diseases, such as AITD, systemic lupus erythematosus, and rheumatoid arthritis (Sardu et al. 2012). Among these, AITDs exhibit a stronger correlation with MG compared to other autoimmune diseases. (Téllez‐Zenteno et al. 2004)

AITDs encompass a group of autoimmune disorders primarily mediated by lymphocytes, representing over 30% of all organ‐specific autoimmune diseases. The main subtypes of AITDs are Graves' disease (GD) and Hashimoto's thyroiditis (HT), with an estimated prevalence of 5%. (Tomer 2014) Clinically, GD is the leading cause of hyperthyroidism, accounting for over 80% of cases (Doubleday and Sippel 2020), while HT is the primary cause of hypothyroidism. Previous research has indicated a higher occurrence of autoimmune thyroid disorders in MG patients compared to other autoimmune diseases (Mao et al. 2011). Clinical trials have also indicated a higher frequency of AITD in MG patients compared to control groups (Weissel, Mayr, and Zeitlhofer 2000). Population‐based cohort studies have suggested a strong correlation between MG and various thyroid diseases, particularly HT (Lin et al. 2017). Similar findings have been reported by Sasivimol Virameteekul and colleagues (Virameteekul et al. 2019). Prior MR analysis have revealed a positive correlation between hypothyroidism and MG (Bao et al. 2024, Li, Ouyang, and Yang 2023), as well as a causal relationship between hyperthyroidism (Li, Ouyang, and Yang 2023), GD (Wang et al. 2024), and MG. MG associated with acetylcholine receptors can be categorized into early‐onset MG and late‐onset MG. Research indicates that early‐onset MG and late‐onset MG exhibit distinct characteristics regarding their association with AITD. The prevalence of AITD is significantly higher in patients with early‐onset MG compared to those with late‐onset MG (Sehgal et al. 2017). Furthermore, early‐onset and late‐onset MG display divergent trends in association with other autoimmune diseases (Maniaol et al. 2012). However, autoimmune diseases are complex and influenced by both genetic and environmental factors, with observational studies susceptible to confounding and reverse causation. Although previous MR analyses have explored the association between AITD and MG, the relationship between AITD and MG subtypes remains underexplored. Thus, we conducted a more comprehensive bidirectional two‐sample MR analyses to investigate the causal relationship between MG, its subtypes, and AITDs, aiming to provide robust evidence for the early prevention of MG and thyroid diseases.

## 2 Materials and Methods

### 2.1 Study Design

**FIGURE 1:**
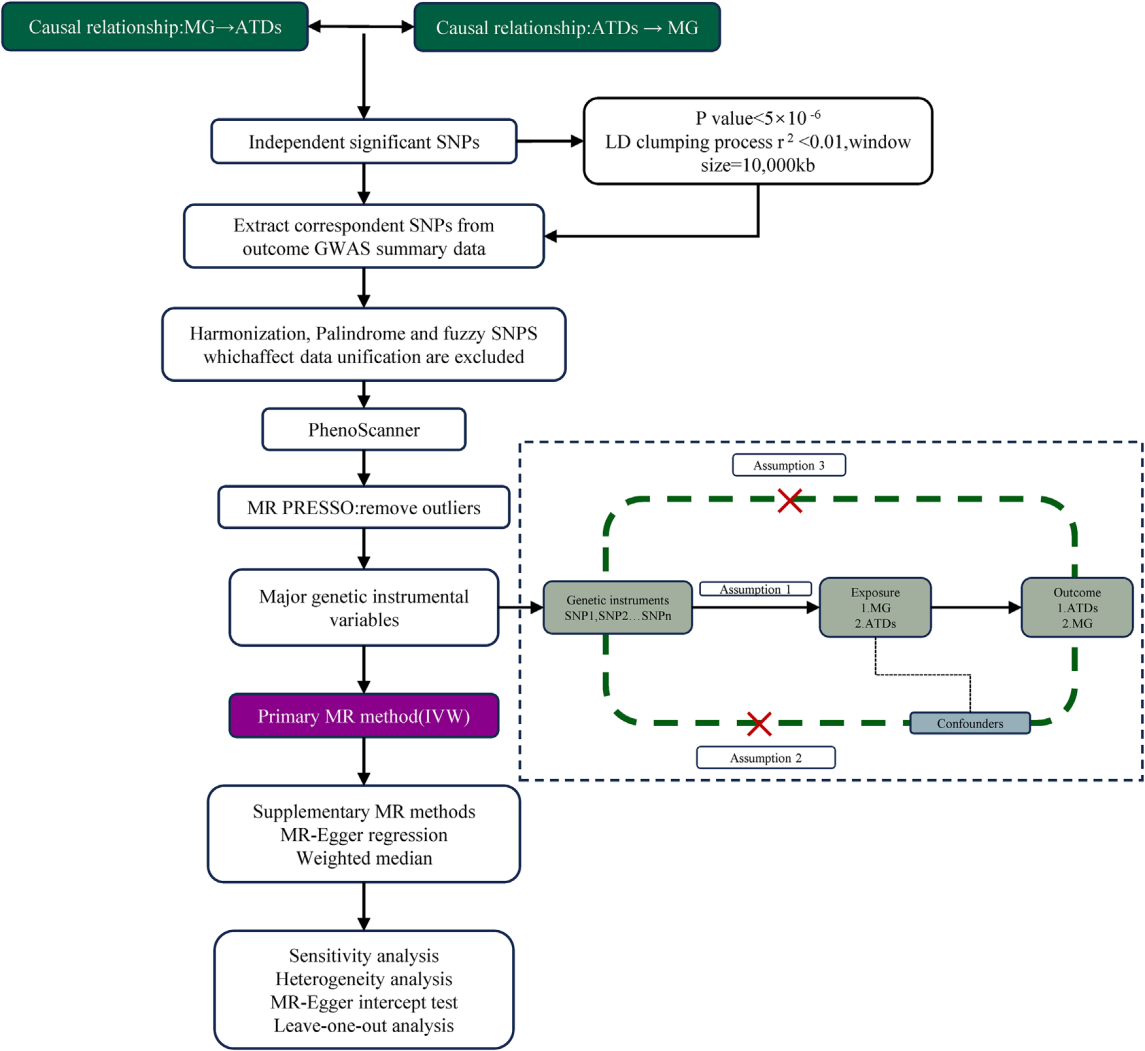
Flowchart of the overall design and hypotheses of the Mendelian randomization analysis.

Figure 1 illustrates the overall research design and fundamental hypotheses of the MR studies. Our investigation employed a two‐sample MR analysis to explore the reciprocal causal links between MG and a spectrum of thyroid diseases, encompassing GD, HT, autoimmune hypothyroidism, and autoimmune hyperthyroidism.

A comprehensive set of sensitivity analyses was conducted to validate the robustness of the findings. The MR analysis rests on three core assumptions, as depicted in Figure 1. Initially, it is crucial that the selected genetic variants used as instrumental variables (IVs) demonstrate a strong and reliable association with the intended exposure. Secondly, these IVs should demonstrate no connection with potential confounding factors. Lastly, the genetic variants should exert an impact on the outcome exclusively through the exposure, signifying a singular causal pathway from the exposure to the outcome devoid of mediation by other factors.

The original studies involved in this research received ethical approvals and participant consents. For our study, we utilized analytical data obtained from publicly accessible databases or previously published studies. Therefore, no additional ethical approvals or participant informed consents were required.

### 2.2 Data Sources

In this investigation, we utilized the extensive genomic data from the largest GWAS, which included a total of 1873 patients diagnosed with MG, matched with 36,370 controls of similar age and sex (Chia et al. 2022). Additionally, our analysis specifically examined summary statistics for two distinct subsets of MG: early‐onset MG (EOMG), comprising 595 patients aged 40 years or younger and 2718 corresponding controls, as well as late‐onset MG (LOMG), consisting of 1278 patients and 33,652 controls. To ensure sample independence, summary statistics for autoimmune hyperthyroidism, autoimmune hypothyroidism, HT, and GD were sourced from the FinnGen database. Specifically, the autoimmune hyperthyroidism GWAS data encompassed 1828 cases and 279,855 controls of European descent, while the autoimmune hypothyroidism data comprised 40,926 cases and 274,069 European descent controls. Additionally, the HT GWAS included 489 cases and 320,703 European descent controls, and the GD data consisted of 2836 cases and 374,441 European descent controls. For further details regarding the incorporated data, please refer to Table 1.

**TABLE 1:** Detailed characterization of GWAS data.

Phenotype	Consortium	Ancestry	Sample size	Web source
Myasthenia gravis	GWAS catalog	European	38,243	(http://ftp.ebi.ac.uk)
EOMG	GWAS catalog	European	3313	—
LOMG	GWAS catalog	European	34,930	—
Hypothyroidism	FINNGen	European	314,995	(https://www.finngen.fi/fi)
Hyperthyroidism	FINNGen	European	281,683	—
Hashimoto thyroiditis	FINNGen	European	321,192	—
Graves' disease	FINNGen	European	377,277	—

### 2.3 Selection of IVs

The process of screening instrumental variables is visually presented in Figure 1. Initially, we identified SNPs that showed significant associations with the exposure (*p* < 5 × 10^−6^). To address potential biases arising from high linkage disequilibrium (LD), we specifically selected SNPs with an LD threshold of *r* ^2^ < 0.01 within a clumping window of 10,000 kb. In order to fulfill the first hypothesis, we applied the Steiger filter to obtain R2 and computed the F‐statistic for each SNP. We excluded weak genetic instruments with an *F*‐statistic <10, thus deriving the average *F*‐value. Additionally, we excluded palindromic and ambiguous SNPs that could affect data consistency (Cheng et al. 2023). Furthermore, aligning with the second major hypothesis, we conducted a thorough search on the Phenoscanner and eliminated SNPs linked to confounding factors associated with autoimmune hypothyroidism such as rheumatoid arthritis, systemic lupus erythematosus, and primary sclerosing cholangitis. Additionally, SNPs associated with risk factors for autoimmune hyperthyroidism, such as GD and levothyroxine sodium treatment, were also excluded. Finally, we utilized MR‐PRESSO analysis to detect and eliminate any outliers. Upon completion of these rigorous selection steps, the finalized SNPs were utilized for subsequent two‐sample MR analyses.

### 2.4 Statistical Analysis

#### 2.4.1 Mendelian Randomization Analysis

In our study, a variety of analytical methods were utilized, with the primary approach being the instrumental variable weighting (IVW) method. This approach assumes that all SNPs are valid genetic instrumental variables. IVW derives an overall estimate of the effect of exposure on the outcome by combining the causal relationship's Wald estimates for each IV (Burgess, Butterworth, and Thompson 2013). To assess the robustness of our results, we also utilized methods such as MR‐Egger regression, and weighted median. We considered a *p* value of <0.012 (0.05/4) to be significantly different after Bonferroni correction, while *p* values between 0.05 and the significant threshold were considered nominally significant.

#### 2.4.2 Sensitivity Analyses

A series of sensitivity analyses were conducted to ascertain the reliability of the causal effect. The MR‐Egger regression method was employed to detect and account for anomalous instrumental variables, providing a consistent estimate of the causal effect uninfluenced by these anomalies (Bowden, Davey Smith, and Burgess 2015). Additionally, MR‐Egger regression was used to assess horizontal pleiotropy, indicated by a *P*‐value < 0.05 for the intercept. This indicates the presence of confounding factors that influence the outcomes through pathways unrelated to the exposure of interest in our study. In such cases, MR‐PRESSO was applied for the identification and adjustment of pleiotropy residuals and outliers (Verbanck et al. 2018). Outliers with pleiotropic effects were excluded, followed by a reassessment of the causal effect. MR‐PRESSO consists of three parts: MR‐PRESSO global test, MR‐PRESSO outlier test, and MR‐PRESSO distortion test. It is a method for detecting and correcting outliers in IVW linear regression (Verbanck et al. 2018).The IVW method with Cochran's Q statistic was employed to assess heterogeneity (Greco et al. 2015), considering a *P*‐value >0.05 as indicative of no heterogeneity. Lastly, a ‘leave‐one‐out’ approach was applied to determine if the exclusion of individual SNPs influenced the effect results. All analyses were conducted using R software (version 4.2.3) and the “Two‐Sample MR” and “MR‐PRESSO” packages for MR analysis.

## 3 Results

### 3.1 Primary Analysis

In our analysis, we employed 28 SNPs as instrumental variables for MG and for hypothyroidism, hyperthyroidism, GD, and HT; we used 355, 33, 35, and 4 SNPs, respectively. All SNPs included in the study demonstrated F‐statistics exceeding 10, indicating a strong association between the selected genetic instrumental variables and MG, while mitigating bias from weak instrumental variables. Detailed characteristics of these genetic instrumental variables are provided in Tables S1 and S2.

**FIGURE 2:**
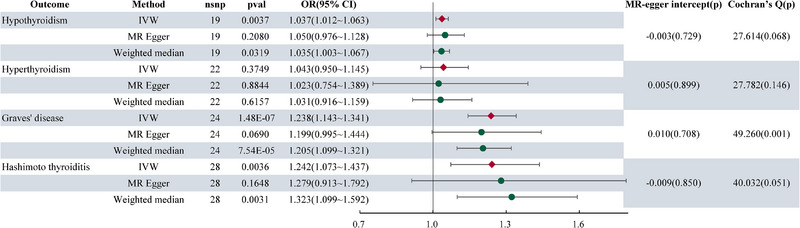
The forest plot showing results from the Mendelian randomization analysis, using MG as exposure to evaluate potential causal associations between MG and AITD.

**FIGURE 3:**
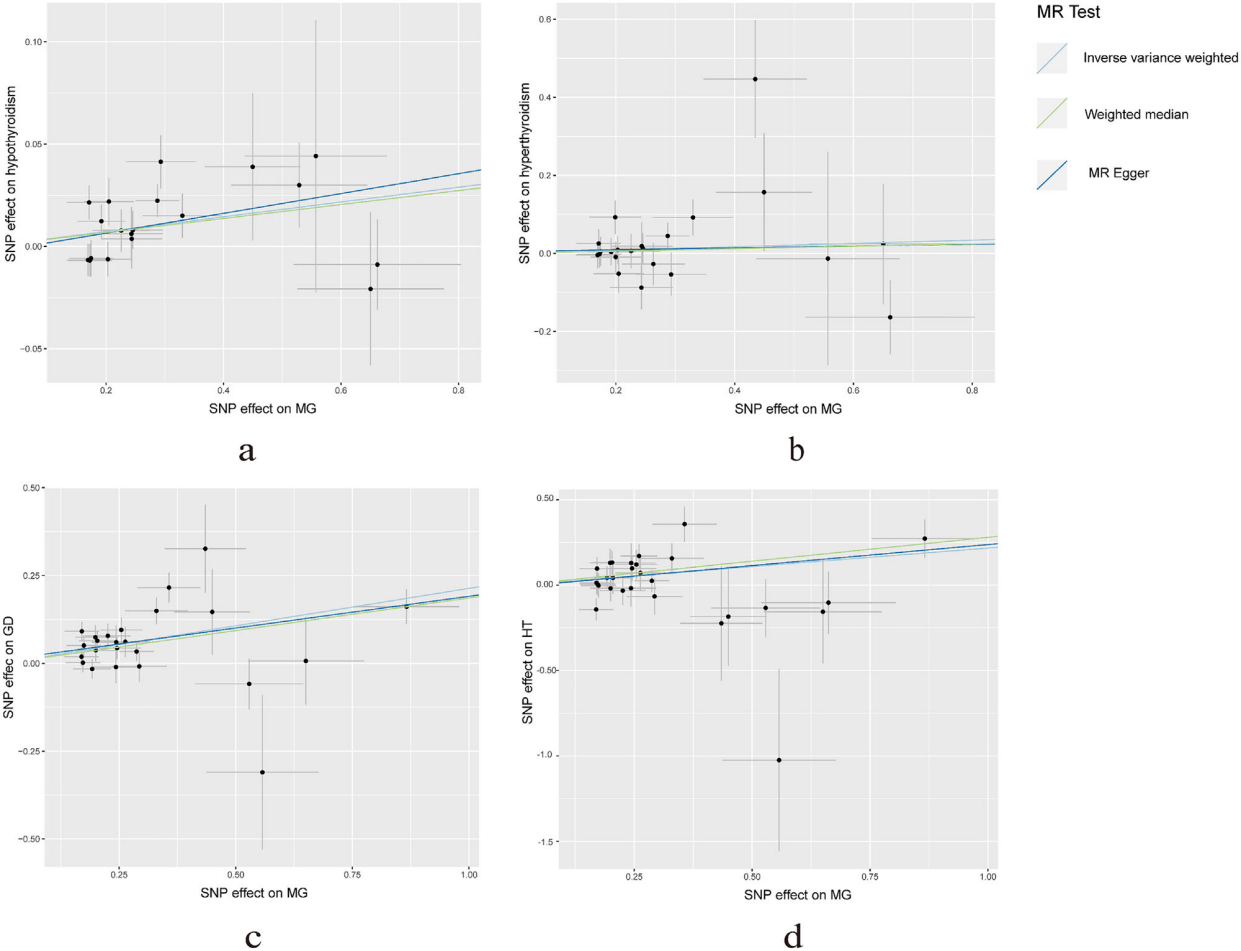
The scatter plot of the causal effect of MG on AITD risk. MG on (a) The scatter plot of the causal effect of MG on autoimmune hypothyroidism risk; (b) The scatter plot of the causal effect of MG on autoimmune hyperthyroidism risk; (c) The scatter plot of the causal effect of MG on Graves' disease risk; (d) The scatter plot of the causal effect of MG on Hashimoto's thyroiditis risk.

The findings of the MR analysis are presented in Figure 2, showcasing the significant associations revealed. Genetically predicted MG demonstrated a notable association with an increased risk of HT (OR = 1.242(1.073–1.437), *P* = 0.0036), as well as with a heightened risk of Grave's disease (OR = 1.238 (1.143–1.341), *P* = 1.48E×10^−7^). Genetically predicted MG was also positively associated with autoimmune hypothyroidism (OR = 1.037 (1.012–1.063), *P* = 0.0037). However, no substantial evidence of association was found between genetically predicted MG and autoimmune hyperthyroidism (OR = 1.043 (0.950–1.145), *P* = 0.3749). Across all analyses, MR‐Egger regression analysis did not detect horizontal pleiotropy. Heterogeneity was only observed in the MR analysis of MG and Graves’ disease. As we adopted the random effects model as our primary analytical method, heterogeneity was deemed acceptable (Burgess et al. 2019). Figure 3 displays the scatter plot for the MR analysis of MG and AITDs. Funnel plot and leave‐one‐out sensitivity analysis revealed no conspicuous abnormalities (Figures S1and S2).

### 3.2 Reverse Analysis

**FIGURE 4:**
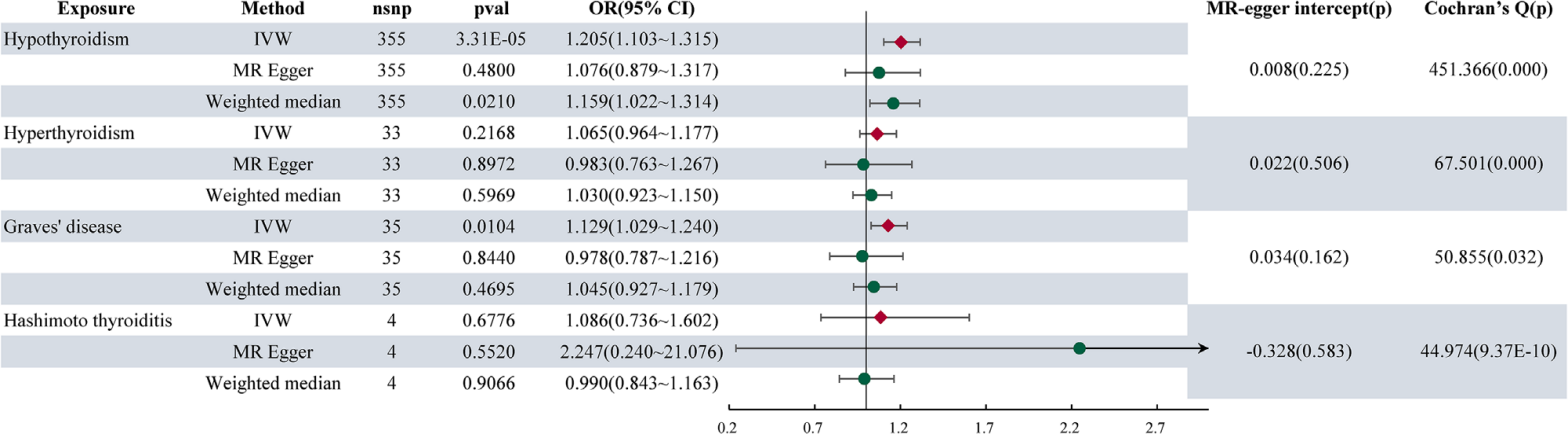
The forest plot showing results from the Mendelian randomization analysis, using MG as the outcome to evaluate potential causal associations between AITDs and MG.

Figure 4 presents the findings of the reverse MR analysis. Our MR analysis revealed a significant link between genetically predicted autoimmune hypothyroidism and an elevated risk of MG (OR = 1.205 (1.103‐1.315), *P* = 3.31E×10^−5^). Furthermore, we identified a notable association between genetically predicted GD and a heightened risk of MG (OR = 1.129 (1.029–1.240), *P* = 0.0104). Conversely, there was no evidence supporting an association between genetically predicted HT and MG, as well as between genetically predicted autoimmune hyperthyroidism and MG. Notably, the MR‐Egger regression analysis did not indicate any evidence of horizontal pleiotropy in the conducted analyses. However, heterogeneity was observed across all analyses, as depicted in Figure 4. Sensitivity analysis revealed no obvious abnormalities in the results. Additionally, Figures S3–S5 present the scatter plot, funnel plot, and leave‐one‐out plot, respectively, complementing the main findings.

### 3.3 Bidirectional Relationship between MG Subgroups and AITD

In examining the relationship between the MG subgroup and AITDs, we discovered a significant association between early‐onset myasthenia gravis (EOMG) and an increased risk of HT (OR (1.073–1.246), *P* = 1.269×10^−4^). The Cochran *Q* test demonstrated no heterogeneity (*P* = 0.067). Contrary to expectations, the reverse analysis did not find an elevated risk of EOMG attributable to HT. Similarly, bidirectional studies exploring the relationship between late‐onset myasthenia gravis (LOMG) and HT did not reveal any significant association. Additionally, conducting a causal analysis on both subgroups, including Grave's disease, autoimmune hyperthyroidism, and autoimmune hypothyroidism, yielded no statistically significant correlation (*p* > 0.05). Notably, MR‐Egger regression analysis across all the examinations failed to detect horizontal pleiotropy. (Table 2)

**TABLE 2:** Univariable Mendelian randomization of EOMG on the risk for AITDs.

Outcome	nSNPs	Method	OR(95%CI)	P
Hypothyroidism	17	IVW	1.01(0.99,1.02)	0.052
		MR Egger	0.99(0.96,1.02)	0.777
		Weighted median	1.01(0.99,1.03)	0.235
Hyperthyroidism	18	IVW	1.00(0.95,1.05)	0.781
		MR Egger	0.90(0.81,1.01)	0.095
		Weighted median	0.99(0.93,1.06)	0.952
Graves' disease	17	IVW	1.00(0.96,1.04)	0.825
		MR Egger	0.99(0.90,1.08)	0.907
		Weighted median	0.98(0.93,1.04)	0.595
Hashimoto thyroiditis	19	IVW	1.15(1.07,1.24)	1.269×10^−4^
		MR Egger	1.22(1.01,1.46)	0.050
		Weighted median	1.18(1.04,1.33)	0.009

## 4 Discussion

Our research has demonstrated a clear association between genetic predisposition to MG and an increased risk of GD, HT, and autoimmune hypothyroidism, while no significant correlation was found with autoimmune hyperthyroidism. In our reverse studies, only autoimmune hypothyroidism and GD emerged as potential risk factors for the development of MG. Furthermore, our bidirectional MR study involving two MG subgroups and four types of AITD unveiled a correlation between EOMG and HT, indicating a heightened risk of HT, although no significant associations were observed with other thyroid conditions in MG subgroups. The robustness of our findings was validated through sensitivity analysis.

Studies have identified genetic overlaps between MG and other autoimmune diseases, with thyroid abnormalities being the most notable (Chia et al. 2022). Earlier MR studies have also highlighted a potential connection between thyroid function and MG (14, 15). A recent MR study (Wang et al. 2024) examined the association between common thyroid diseases and MG, and similar conclusions were drawn regarding the association between MG and DG. However, the study did not report on the relationship between MG subtypes and AITD. Additionally, the associations between hyperthyroidism, HT, and MG remain unreported. Our study conducted a more comprehensive analysis of the relationships among hyperthyroidism, HT, and MG. By differentiating between early‐onset and late‐onset MG in conjunction with AITD, we enhanced the investigation of the relationship between MG subtypes and AITD, yielding the anticipated results. Beyond genetic studies, a substantial body of clinical research provides evidence for the relationship between the two. (Tamer et al. 2016) observed that 24% of MG patients had comorbid autoimmune diseases, with half of them (12 patients) having AITDs, including 9 with HT and 3 with GD. It is of utmost importance to emphasize that the number of diagnoses for AITDs did not significantly differ before and after the onset of MG. Moreover, another study provided compelling evidence supporting the coexistence of MG and AITD, indicating a higher prevalence of GD, HT, and autoimmune hyperthyroidism in MG. This suggests that MG may potentially act as a risk factor for AITD (Song et al. 2019).

In previous research, the relationship between various subtypes of thyroid disease and MG has been extensively studied. Amin et al. reported that GD is more prevalent in MG patients compared to HT among all thyroid diseases (Amin et al. 2020). Nikolina et al. discovered a stronger correlation between MG and hypothyroidism (Tanovska et al. 2018), which aligns with our findings. However, another study reached the opposite conclusion (Salhi and Ajdi 2019), suggesting a higher prevalence of the association between hyperthyroidism and MG compared to hypothyroidism. Concerning the reverse association between AITDs and MG, related studies have indicated a 186% increased risk of AITDs in MG patients, while the prevalence of thyroid disease combined with MG varies across existing studies (Song et al. 2019). Epidemiological studies have revealed that approximately 5‐10% of MG patients will develop AITD, whereas the occurrence of MG in AITD patients is notably low (0.2%) (Yaman and Yaman 2003). This disparity may be attributed to a combination of factors, including genetics, environment, diagnostic criteria for thyroid diseases, sample size, and study population. To validate our findings, it is imperative to carry out thorough prospective cohort studies in the future, considering the limited research available on the subject. These studies would allow for a comprehensive evaluation of the relationship between MG and AITDs.

IgG antibodies are crucial in various autoimmune diseases, including MG, GD (McIver and Morris 1998), and thyroid ophthalmopathy (Men, Kossler, and Wester 2021). However, the role of IgG antibody‐mediated autoimmunity in MG and AITD is not yet fully understood. Specifically, the mechanisms behind these conditions remain unclear, particularly since a small number of MG patients have not exhibited detectable autoantibodies. For the majority of antibody‐positive MG patients, previous research has introduced new perspectives on treatment strategies for MG combined with thyroid disease. The neonatal Fc receptor (FcRn), an MHC Class I‐like molecule, is vital for maintaining IgG levels (Ober et al. 2004). Targeted inhibition of FcRn to control IgG levels has emerged as a potential mechanism for developing MG therapeutics, such as niparalizumab. Drugs like rozanolizumab, IMVT‐1401, orilanolimab, and efgartigimod have demonstrated potential in reducing IgG levels (Wolfe et al. 2021). In the context of thyroid disease, a proof‐of‐concept randomized controlled trial (Kahaly et al. 2023) indicated that weekly subcutaneous administration of bartolizumab (680 mg) for 12 weeks improved both the GO‐QoL total score and the appearance subscale. Additionally, another randomized controlled trial (Wolf et al. 2023) showed that a novel monoclonal antibody targeting FcRn significantly reduced thyroid‐stimulating hormone receptor antibodies (TSH‐R‐Ab) in GD patients. Although evidence is still lacking regarding the efficacy of simultaneous intervention for MG and thyroid disease, these studies offer new insights into co‐intervention therapy.

The correlation between MG and thyroid diseases may be influenced by a shared genetic background, such as the histocompatibility complex commonly known as the human leukocyte antigen or (HLA). The HLA sites are recognized as the primary susceptibility sites for MG. Studies have clarified the association between HLA class I and II genes and MG, and it has been established that the earliest known susceptibility genes for AITDs are also MHC class II genes, including the HLA‐DR3 antigen associated with GD. HLA haplotypes play a role in the development of MG. Additionally, patients with autoimmune diseases may have specific alleles known to be associated with reduced immune regulatory function. The influence of genetic factors between these two diseases, regardless of their subtypes, warrants further investigation.

GD and HT are characterized by thyrotoxicosis and hypothyroidism, respectively. GD stands as the predominant cause of persistent hyperthyroidism in adults, comprising approximately 80% to 85% of total cases (Burch and Cooper 2015). Although GD and HT have distinct impacts on thyroid function, they share a common immune pathogenic mechanism characterized by lymphocyte infiltration and the production of thyroid autoantibodies. The underlying pathogenesis of MG remains unclear, but it is known to be closely associated with thymic abnormalities, immune regulatory deficiencies, and sex hormone influences. The mechanisms involved may vary across different subtypes of MG.

The immune system functions within a tightly regulated framework that depends on a delicate balance between pro‐inflammatory and anti‐inflammatory signals, as well as cellular responses. Specific Th17 and Treg cells, along with their associated cytokines, are crucial in maintaining this balance. In particular, Th17 cells and their characteristic cytokine IL‐17 exhibit a close connection to the pathogenesis of HT and GD (Bossowski et al. 2012). Furthermore, aberrant expression of Treg cells has been observed in patients with AITDs (Vitales‐Noyola et al. 2018). Studies have demonstrated that IL‐21 plays a pivotal role in promoting the differentiation of CD4+ T cells into Th17 cells while simultaneously inhibiting the differentiation of Treg cells. This activation of subsequent immune responses contributes to the development of Graves' disease (Tan et al. 2019). In addition to its impact on T cell structure and function, dysfunctional thymic activity also leads to elevated expression levels of inflammatory cytokines like IL‐6, IL‐1, interferon (IFN), and tumor necrosis factor (TNF). Additionally, acetylcholine receptor antibodies are the most prevalent pathogenic antibodies in MG (Mantegazza, Bernasconi, and Cavalcante 2018), with the thymus serving as a crucial site for their production (Grob et al. 2008). In summary, abnormalities in T cell development and function mediate the connection between MG and AITDs. Further investigation is necessary to explore potential additional mechanisms of comorbidity.

Our research possesses several advantages that contribute to its strength. Firstly, we employed MR research methods, effectively minimizing inherent confounding factors and biases typically encountered in observational studies. Secondly, we conducted a comprehensive analysis of the association between MG and AITDs by utilizing a bidirectional MR approach, thereby addressing the limitations of previous MR analyses. Lastly, our research findings establish a bidirectional relationship between MG and AITDs, providing evidence‐based support for the bidirectional prevention of both conditions. Nonetheless, this study does have certain limitations. Firstly, as our analysis is based on GWAS, the genetic instrumental variables used are derived from individuals of European descent. Consequently, the generalizability of our results to other ethnicities remains uncertain. Secondly, the genetic databases utilized for MG and AITDs in our study lack subgroup‐specific information such as age, sex, and pathogenic antibodies. Since various antibodies are associated with distinct genetic susceptibilities, and the incidence of AITDs varies by sex, our study was unable to carry out subgroup analysis to account for the confounding factors that may arise from these aspects.

## 5 Conclusion

Our research suggested the clinical importance of considering the possibility of comorbidities, advocating for appropriate routine screening to facilitate early prevention, detection, and treatment. Further research is needed to investigate the complex mechanisms of this interrelationship, which could provide potential target genes and pathways for future disease treatment. More rigorous clinical studies, advanced research methods, and efficient multidisciplinary collaborations may play a crucial role in future discoveries.

## Author Contributions

**Yao Wang**: Conceptualization, writing–original draft. **Ke Wang**: Conceptualization; writing–original draft. **Jing Lu**: Formal analysis. **Peng Xu**: Visualization. **Dongmei Zhang**: Visualization. **Xinzhi Chen**: Conceptualization. **Jian Wang**: Writing–review and editing.

## Conflicts of Interest

The authors declare no conflicts of interest.

### Peer Review

The peer review history for this article is available at https://publons.com/publon/10.1002/brb3.70235.

## Ethics Statement

The use of publicly available data in this study eliminates the need for an additional ethical declaration regarding human and animal rights. It should be noted that all studies accessed in the present research were approved by their respective ethics committees.

## Supporting information



Supplementary Table S1: Characteristics of Genetic Instrumental Variables in Univariate Mendelian Randomization Analyses of myasthenia gravis.
Supplementary Table 2: Characteristics of Genetic Instrumental Variables in Univariate Mendelian Randomization Analyses of autoimmune thyroid disease.
Supplementary FigureS1 Funnel plots of the causal effect of MG on AITD. (a) Funnel plot of the causal effect of MG on autoimmune hypothyroidism; (b) Funnel plot of the causal effect of MG on autoimmune hyperthyroidism; (c) Funnel plot of the causal effect of MG on Graves' disease; (d) Funnel plot of the causal effect of MG on Hashimoto's thyroiditis.
Supplementary FigureS2 Leave‐one‐out analysis of the causal effect of MG on AITD. (a) Leave‐one‐out analysis of the causal effect of MG on autoimmune hypothyroidism; (b) Leave‐one‐out analysis of the causal effect of MG on autoimmune hyperthyroidism; (c) Leave‐one‐out analysis of the causal effect of MG on Graves' disease; (d) Leave‐one‐out analysis of the causal effect of MG on Hashimoto's thyroiditis.
Supplementary FigureS3 Scatter plots of the causal effect of AITD on MG. (a) Scatter plot of the causal effect of autoimmune hypothyroidism on MG; (b) Scatter plot of the causal effect of autoimmune hyperthyroidism on MG; (c) Scatter plot of the causal effect of Graves' disease on MG; (d) Scatter plot of the causal effect of Hashimoto's thyroiditis on MG.
Supplementary FigureS4 Funnel plots of the causal effect of AITD on MG. (a) Funnel plot of the causal effect of autoimmune hypothyroidism on MG; (b) Funnel plot of the causal effect of autoimmune hyperthyroidism on MG; (c) Funnel plot of the causal effect of Graves' disease on MG; (d) Funnel plot of the causal effect of Hashimoto's thyroiditis on MG.
Supplementary FigureS5 Leave‐one‐out plots of the causal effect of AITD on MG. (a) Leave‐one‐out plot of the causal effect of autoimmune hypothyroidism on MG; (b) Leave‐one‐out plot of the causal effect of autoimmune hyperthyroidism on MG; (c) Leave‐one‐out plot of the causal effect of Graves' disease on MG; (d) Leave‐one‐out plot of the causal effect of Hashimoto's thyroiditis on MG.

## Data Availability

The data pertaining to AITD presented in this study can be accessed through the publicly available FinnGen Consortium [https://www.finngen.fi/en]. Additionally, the GWAS summary data for myasthenia gravis can be downloaded from the GWAS Catalog database (http://ftp.ebi.ac.uk).
