# Supplementary material for: Association of Myasthenia Gravis With Autoimmune Thyroid Disease: A Bidirectional Mendelian Randomization Study
Source: Brain Behav. 2025 Jan 19;15(1):e70235. doi: 10.1002/brb3.70235 (PMC11743983; doi:10.1002/brb3.70235)
Supplement: Supplementary file 1 — Supplementary Table S1: Characteristics of Genetic Instrumental Variables in Univariate Mendelian Randomization Analyses of myasthenia gravis. Supplementary Table 2: Characteristics of Genetic Instrumental Variables in Univariate Mendelian Randomization Analyses of autoimmune thyroid disease. Supplementary FigureS1 Funnel plots of the causal effect of MG on AITD. (a) Funnel plot of the causal effect of MG on autoimmune hypothyroidism; (b) Funnel plot of the causal effect of MG on autoimmune hyperthyroidism; (c) Funnel plot of the causal effect of MG on Graves' disease; (d) Funnel plot of the causal effect of MG on Hashimoto's thyroiditis. Supplementary FigureS2 Leave‐one‐out analysis of the causal effect of MG on AITD. (a) Leave‐one‐out analysis of the causal effect of MG on autoimmune hypothyroidism; (b) Leave‐one‐out analysis of the causal effect of MG on autoimmune hyperthyroidism; (c) Leave‐one‐out analysis of the causal effect of MG on Graves' disease; (d) Leave‐one‐out analysis of the causal effect of MG on Hashimoto's thyroiditis. Supplementary FigureS3 Scatter plots of the causal effect of AITD on MG. (a) Scatter plot of the causal effect of autoimmune hypothyroidism on MG; (b) Scatter plot of the causal effect of autoimmune hyperthyroidism on MG; (c) Scatter plot of the causal effect of Graves' disease on MG; (d) Scatter plot of the causal effect of Hashimoto's thyroiditis on MG. Supplementary FigureS4 Funnel plots of the causal effect of AITD on MG. (a) Funnel plot of the causal effect of autoimmune hypothyroidism on MG; (b) Funnel plot of the causal effect of autoimmune hyperthyroidism on MG; (c) Funnel plot of the causal effect of Graves' disease on MG; (d) Funnel plot of the causal effect of Hashimoto's thyroiditis on MG. Supplementary FigureS5 Leave‐one‐out plots of the causal effect of AITD on MG. (a) Leave‐one‐out plot of the causal effect of autoimmune hypothyroidism on MG; (b) Leave‐one‐out plot of the causal effect of autoimmune hyperthyroidism on [file BRB3-15-e70235-s001.docx]

**Supplementary Table 1**: Characteristics of Genetic Instrumental Variables in Univariate Mendelian Randomisation Analyses of myasthenia gravis.

|  | SNP | EA | OA | BETA | se | pval |
| --- | --- | --- | --- | --- | --- | --- |
| 1 | rs111263101 | G | A | 0.2048 | 0.0425 | 1.44E-06 |
| 2 | rs112477645 | A | G | -0.2931 | 0.0592 | 7.40E-07 |
| 3 | rs116976127 | T | G | 0.5287 | 0.1157 | 4.91E-06 |
| 4 | rs11985475 | T | C | 0.1724 | 0.0375 | 4.23E-06 |
| 5 | rs12637233 | A | G | 0.2436 | 0.052 | 2.82E-06 |
| 6 | rs12884697 | G | A | 0.2632 | 0.0528 | 6.23E-07 |
| 7 | rs12901830 | T | G | -0.2455 | 0.0518 | 2.17E-06 |
| 8 | rs138255937 | T | C | 0.5571 | 0.1206 | 3.86E-06 |
| 9 | rs147362774 | C | T | 0.6502 | 0.1248 | 1.88E-07 |
| 10 | rs17172212 | T | C | 0.6619 | 0.1426 | 3.44E-06 |
| 11 | rs2523595 | G | A | 0.2602 | 0.039 | 2.55E-11 |
| 12 | rs28362679 | A | G | 0.4347 | 0.0869 | 5.70E-07 |
| 13 | rs28597864 | C | T | 0.1998 | 0.0425 | 2.56E-06 |
| 14 | rs35274388 | A | G | 0.4495 | 0.0812 | 3.07E-08 |
| 15 | rs35695082 | C | T | 0.2257 | 0.0487 | 3.54E-06 |
| 16 | rs3809717 | A | C | 0.2031 | 0.0394 | 2.63E-07 |
| 17 | rs4377259 | C | T | 0.1921 | 0.0408 | 2.46E-06 |
| 18 | rs4409785 | C | T | 0.2545 | 0.045 | 1.54E-08 |
| 19 | rs4574025 | C | T | 0.2874 | 0.0369 | 7.09E-15 |
| 20 | rs560607175 | A | C | -0.3568 | 0.0679 | 1.50E-07 |
| 21 | rs56074046 | A | G | 0.1743 | 0.0372 | 2.87E-06 |
| 22 | rs6508442 | T | C | 0.1711 | 0.0374 | 4.62E-06 |
| 23 | rs6590685 | C | A | 0.1693 | 0.0368 | 4.14E-06 |
| 24 | rs6819946 | T | C | -0.17 | 0.0368 | 3.75E-06 |
| 25 | rs76815088 | C | T | -0.8661 | 0.1128 | 1.58E-14 |
| 26 | rs7727530 | A | C | -0.3299 | 0.0675 | 1.04E-06 |
| 27 | rs7869982 | A | G | 0.2431 | 0.0531 | 4.64E-06 |
| 28 | rs912425 | G | A | -0.199 | 0.0436 | 4.98E-06 |

**Supplementary Table 2**: Characteristics of Genetic Instrumental Variables in Univariate Mendelian Randomisation Analyses of autoimmune thyroid disease.

| Trait | SNP | EA | OA | BETA | se | pval |
| --- | --- | --- | --- | --- | --- | --- |
| Hypothyroidism | rs10035379 | A | G | -0.04251 | 0.007945 | 8.75E-08 |
| Hypothyroidism | rs10055404 | G | A | -0.05069 | 0.008074 | 3.44E-10 |
| Hypothyroidism | rs10116520 | G | A | 0.09222 | 0.007961 | 4.99E-31 |
| Hypothyroidism | rs10118880 | A | G | -0.05997 | 0.008713 | 5.89E-12 |
| Hypothyroidism | rs10122640 | A | G | 0.091196 | 0.016641 | 4.25E-08 |
| Hypothyroidism | rs10125995 | T | C | -0.04428 | 0.007844 | 1.65E-08 |
| Hypothyroidism | rs10166287 | G | T | -0.09197 | 0.013121 | 2.39E-12 |
| Hypothyroidism | rs10177852 | C | T | 0.053798 | 0.009716 | 3.08E-08 |
| Hypothyroidism | rs10259879 | G | A | 0.06374 | 0.009285 | 6.65E-12 |
| Hypothyroidism | rs10277273 | G | T | -0.04025 | 0.008738 | 4.09E-06 |
| Hypothyroidism | rs10403662 | T | G | -0.04262 | 0.009147 | 3.16E-06 |
| Hypothyroidism | rs10414260 | G | A | 0.051384 | 0.009493 | 6.20E-08 |
| Hypothyroidism | rs10416358 | G | A | 0.079297 | 0.008575 | 2.31E-20 |
| Hypothyroidism | rs10425559 | G | A | -0.05543 | 0.007987 | 3.93E-12 |
| Hypothyroidism | rs10474556 | T | C | 0.046078 | 0.008491 | 5.75E-08 |
| Hypothyroidism | rs10514934 | C | T | -0.08232 | 0.011099 | 1.20E-13 |
| Hypothyroidism | rs10740068 | C | A | -0.05962 | 0.008111 | 1.97E-13 |
| Hypothyroidism | rs10748781 | A | C | -0.07843 | 0.008206 | 1.20E-21 |
| Hypothyroidism | rs10753774 | T | C | 0.04209 | 0.008025 | 1.56E-07 |
| Hypothyroidism | rs10762399 | A | G | 0.040978 | 0.008819 | 3.38E-06 |
| Hypothyroidism | rs10814915 | C | T | -0.07083 | 0.007829 | 1.48E-19 |
| Hypothyroidism | rs10817674 | A | C | 0.049196 | 0.00797 | 6.71E-10 |
| Hypothyroidism | rs10818050 | G | A | 0.24571 | 0.008363 | 9.33E-190 |
| Hypothyroidism | rs10818903 | T | G | -0.04135 | 0.007964 | 2.07E-07 |
| Hypothyroidism | rs10823620 | G | T | 0.036895 | 0.007873 | 2.78E-06 |
| Hypothyroidism | rs10839472 | T | C | 0.042808 | 0.008555 | 5.62E-07 |
| Hypothyroidism | rs10849345 | G | A | 0.042863 | 0.008929 | 1.58E-06 |
| Hypothyroidism | rs10917469 | G | A | -0.12112 | 0.012245 | 4.54E-23 |
| Hypothyroidism | rs10973773 | G | A | 0.041106 | 0.008665 | 2.10E-06 |
| Hypothyroidism | rs11079035 | A | G | 0.075624 | 0.009635 | 4.20E-15 |
| Hypothyroidism | rs111352680 | A | G | -0.05506 | 0.008447 | 7.13E-11 |
| Hypothyroidism | rs11183198 | C | T | -0.0619 | 0.011514 | 7.63E-08 |
| Hypothyroidism | rs111937080 | C | T | 0.160526 | 0.022336 | 6.63E-13 |
| Hypothyroidism | rs11204666 | T | C | -0.06184 | 0.011563 | 8.90E-08 |
| Hypothyroidism | rs112502960 | A | G | 0.036486 | 0.007889 | 3.75E-06 |
| Hypothyroidism | rs11259123 | T | C | -0.04409 | 0.008197 | 7.49E-08 |
| Hypothyroidism | rs11259262 | C | T | 0.050261 | 0.010801 | 3.26E-06 |
| Hypothyroidism | rs113524864 | T | C | 0.117701 | 0.019339 | 1.16E-09 |
| Hypothyroidism | rs115548871 | T | G | -0.16494 | 0.031314 | 1.38E-07 |
| Hypothyroidism | rs11611847 | C | T | -0.07182 | 0.014334 | 5.42E-07 |
| Hypothyroidism | rs116334589 | T | C | -0.14551 | 0.031359 | 3.48E-06 |
| Hypothyroidism | rs116909374 | T | C | -0.21252 | 0.023968 | 7.53E-19 |
| Hypothyroidism | rs11699804 | C | T | -0.05681 | 0.010858 | 1.67E-07 |
| Hypothyroidism | rs117276254 | G | A | 0.159656 | 0.03394 | 2.55E-06 |
| Hypothyroidism | rs117362948 | A | G | 0.200248 | 0.039708 | 4.58E-07 |
| Hypothyroidism | rs117643807 | T | C | 0.112444 | 0.016358 | 6.25E-12 |
| Hypothyroidism | rs118015935 | T | C | -0.10436 | 0.020247 | 2.54E-07 |
| Hypothyroidism | rs118036720 | C | T | 0.189729 | 0.037838 | 5.32E-07 |
| Hypothyroidism | rs11822254 | G | A | -0.05221 | 0.008217 | 2.10E-10 |
| Hypothyroidism | rs11830037 | A | C | 0.099683 | 0.013605 | 2.35E-13 |
| Hypothyroidism | rs11897732 | A | G | 0.046327 | 0.007975 | 6.29E-09 |
| Hypothyroidism | rs11919503 | C | T | -0.04872 | 0.010174 | 1.68E-06 |
| Hypothyroidism | rs11935941 | C | A | -0.14146 | 0.012395 | 3.59E-30 |
| Hypothyroidism | rs11969311 | C | A | -0.05691 | 0.009033 | 2.97E-10 |
| Hypothyroidism | rs11998914 | A | G | 0.056352 | 0.008169 | 5.27E-12 |
| Hypothyroidism | rs1203940 | C | T | 0.102604 | 0.009796 | 1.13E-25 |
| Hypothyroidism | rs12136119 | T | C | -0.06538 | 0.008894 | 1.97E-13 |
| Hypothyroidism | rs12174306 | T | C | -0.14416 | 0.018332 | 3.72E-15 |
| Hypothyroidism | rs12200852 | T | C | 0.050691 | 0.008377 | 1.44E-09 |
| Hypothyroidism | rs12347187 | G | A | 0.05061 | 0.009084 | 2.52E-08 |
| Hypothyroidism | rs12348448 | G | A | -0.04396 | 0.008284 | 1.12E-07 |
| Hypothyroidism | rs1239704 | A | G | -0.0513 | 0.008457 | 1.31E-09 |
| Hypothyroidism | rs12427225 | G | A | -0.10501 | 0.021417 | 9.43E-07 |
| Hypothyroidism | rs12483240 | T | C | -0.07331 | 0.016002 | 4.63E-06 |
| Hypothyroidism | rs12493446 | T | C | 0.041829 | 0.008404 | 6.46E-07 |
| Hypothyroidism | rs12548000 | C | T | -0.04458 | 0.009704 | 4.34E-06 |
| Hypothyroidism | rs12550413 | T | C | 0.057381 | 0.009525 | 1.70E-09 |
| Hypothyroidism | rs12697352 | A | G | -0.04994 | 0.008039 | 5.24E-10 |
| Hypothyroidism | rs12736474 | A | G | 0.049497 | 0.008437 | 4.45E-09 |
| Hypothyroidism | rs12756019 | A | G | -0.05763 | 0.008017 | 6.53E-13 |
| Hypothyroidism | rs1277018 | G | A | 0.054818 | 0.009211 | 2.66E-09 |
| Hypothyroidism | rs12786130 | T | C | -0.04386 | 0.009417 | 3.20E-06 |
| Hypothyroidism | rs1284567 | G | A | 0.038746 | 0.007862 | 8.29E-07 |
| Hypothyroidism | rs12865518 | A | G | 0.045816 | 0.009285 | 8.03E-07 |
| Hypothyroidism | rs12922725 | G | A | -0.04949 | 0.008879 | 2.49E-08 |
| Hypothyroidism | rs12923006 | A | G | 0.072125 | 0.010099 | 9.23E-13 |
| Hypothyroidism | rs12967678 | A | G | 0.074429 | 0.012017 | 5.88E-10 |
| Hypothyroidism | rs13105678 | A | C | 0.042807 | 0.008028 | 9.71E-08 |
| Hypothyroidism | rs13132077 | C | T | 0.038271 | 0.00808 | 2.18E-06 |
| Hypothyroidism | rs13137589 | G | A | -0.06319 | 0.008187 | 1.17E-14 |
| Hypothyroidism | rs13145548 | G | A | -0.03907 | 0.007963 | 9.31E-07 |
| Hypothyroidism | rs1317983 | C | T | 0.102264 | 0.008506 | 2.69E-33 |
| Hypothyroidism | rs1319091 | A | C | -0.05591 | 0.00969 | 7.93E-09 |
| Hypothyroidism | rs13379670 | C | T | 0.044045 | 0.00928 | 2.07E-06 |
| Hypothyroidism | rs13447704 | C | T | -0.1411 | 0.024805 | 1.28E-08 |
| Hypothyroidism | rs1371867 | C | A | 0.0367 | 0.007898 | 3.37E-06 |
| Hypothyroidism | rs137593 | T | C | -0.04017 | 0.007831 | 2.90E-07 |
| Hypothyroidism | rs138014877 | C | T | -0.12846 | 0.025047 | 2.92E-07 |
| Hypothyroidism | rs139717717 | G | A | -0.22534 | 0.044069 | 3.17E-07 |
| Hypothyroidism | rs140932965 | T | C | -0.1539 | 0.031461 | 1.00E-06 |
| Hypothyroidism | rs141281927 | A | G | 0.116757 | 0.020166 | 7.05E-09 |
| Hypothyroidism | rs141686764 | G | A | -0.16437 | 0.02976 | 3.33E-08 |
| Hypothyroidism | rs1420343 | G | A | 0.038953 | 0.007965 | 1.01E-06 |
| Hypothyroidism | rs142647938 | A | C | -0.15445 | 0.027075 | 1.17E-08 |
| Hypothyroidism | rs143278243 | A | G | 0.195821 | 0.026539 | 1.60E-13 |
| Hypothyroidism | rs143402259 | A | G | -0.22012 | 0.044977 | 9.88E-07 |
| Hypothyroidism | rs143505063 | T | C | -0.15914 | 0.033509 | 2.04E-06 |
| Hypothyroidism | rs144101845 | A | C | 0.084827 | 0.015572 | 5.11E-08 |
| Hypothyroidism | rs1441172 | T | C | -0.05256 | 0.008527 | 7.06E-10 |
| Hypothyroidism | rs144287831 | C | T | -0.03997 | 0.008576 | 3.16E-06 |
| Hypothyroidism | rs144651842 | A | G | 0.114059 | 0.014513 | 3.87E-15 |
| Hypothyroidism | rs145353654 | G | A | 0.080571 | 0.015 | 7.82E-08 |
| Hypothyroidism | rs146158994 | A | G | -0.07948 | 0.016712 | 1.98E-06 |
| Hypothyroidism | rs146811683 | C | T | 0.159612 | 0.034588 | 3.94E-06 |
| Hypothyroidism | rs146869774 | G | A | -0.053 | 0.010488 | 4.34E-07 |
| Hypothyroidism | rs146900614 | G | A | 0.136228 | 0.027817 | 9.71E-07 |
| Hypothyroidism | rs147791594 | A | C | 0.132104 | 0.023734 | 2.61E-08 |
| Hypothyroidism | rs147857072 | C | T | -0.19456 | 0.035411 | 3.92E-08 |
| Hypothyroidism | rs148076190 | G | A | 0.129453 | 0.028143 | 4.23E-06 |
| Hypothyroidism | rs149457045 | A | G | -0.20331 | 0.033774 | 1.75E-09 |
| Hypothyroidism | rs150524936 | C | T | -0.11748 | 0.023154 | 3.90E-07 |
| Hypothyroidism | rs150724213 | A | G | 0.120733 | 0.023273 | 2.13E-07 |
| Hypothyroidism | rs151102833 | T | C | -0.04883 | 0.010551 | 3.69E-06 |
| Hypothyroidism | rs1534424 | C | A | -0.05633 | 0.008008 | 1.99E-12 |
| Hypothyroidism | rs1672989 | T | C | -0.06299 | 0.013587 | 3.55E-06 |
| Hypothyroidism | rs17024506 | C | T | -0.15126 | 0.032958 | 4.45E-06 |
| Hypothyroidism | rs1709772 | A | G | 0.038653 | 0.007942 | 1.13E-06 |
| Hypothyroidism | rs17364832 | G | T | 0.078807 | 0.008543 | 2.85E-20 |
| Hypothyroidism | rs1739124 | G | A | 0.052419 | 0.011315 | 3.61E-06 |
| Hypothyroidism | rs1739865 | G | T | 0.045965 | 0.008836 | 1.97E-07 |
| Hypothyroidism | rs1751870 | C | T | -0.06045 | 0.012744 | 2.10E-06 |
| Hypothyroidism | rs17709152 | A | G | 0.096283 | 0.020581 | 2.89E-06 |
| Hypothyroidism | rs17741863 | G | A | -0.07191 | 0.015709 | 4.70E-06 |
| Hypothyroidism | rs17875410 | T | C | -0.07164 | 0.014284 | 5.29E-07 |
| Hypothyroidism | rs1808192 | G | A | -0.04599 | 0.007974 | 8.01E-09 |
| Hypothyroidism | rs182874419 | C | T | 0.082582 | 0.017819 | 3.58E-06 |
| Hypothyroidism | rs1884447 | A | G | 0.037554 | 0.007915 | 2.09E-06 |
| Hypothyroidism | rs1885013 | A | G | 0.050481 | 0.008527 | 3.22E-09 |
| Hypothyroidism | rs188740215 | C | T | 0.102539 | 0.02193 | 2.93E-06 |
| Hypothyroidism | rs1930438 | C | T | -0.04648 | 0.009923 | 2.81E-06 |
| Hypothyroidism | rs194729 | T | C | -0.04006 | 0.008199 | 1.03E-06 |
| Hypothyroidism | rs1969016 | G | T | 0.046183 | 0.009143 | 4.40E-07 |
| Hypothyroidism | rs1990760 | T | C | 0.063818 | 0.007959 | 1.07E-15 |
| Hypothyroidism | rs200383 | C | T | -0.04478 | 0.009205 | 1.15E-06 |
| Hypothyroidism | rs201619070 | T | C | -0.04296 | 0.008438 | 3.55E-07 |
| Hypothyroidism | rs2029818 | C | T | 0.041014 | 0.007863 | 1.83E-07 |
| Hypothyroidism | rs2046045 | G | T | 0.127336 | 0.007899 | 1.81E-58 |
| Hypothyroidism | rs2049218 | T | C | -0.10277 | 0.008075 | 4.19E-37 |
| Hypothyroidism | rs212388 | T | C | 0.05037 | 0.008277 | 1.16E-09 |
| Hypothyroidism | rs2124594 | C | T | -0.05939 | 0.008461 | 2.23E-12 |
| Hypothyroidism | rs2243540 | G | A | 0.057775 | 0.011909 | 1.23E-06 |
| Hypothyroidism | rs2246704 | C | T | 0.039692 | 0.008019 | 7.42E-07 |
| Hypothyroidism | rs2275710 | T | C | 0.066192 | 0.007852 | 3.45E-17 |
| Hypothyroidism | rs2336166 | G | A | -0.03827 | 0.008192 | 2.98E-06 |
| Hypothyroidism | rs235220 | T | C | -0.04954 | 0.009717 | 3.43E-07 |
| Hypothyroidism | rs244073 | T | G | 0.079578 | 0.017379 | 4.67E-06 |
| Hypothyroidism | rs2442752 | C | T | 0.127725 | 0.008143 | 1.90E-55 |
| Hypothyroidism | rs244687 | G | A | -0.07411 | 0.008934 | 1.08E-16 |
| Hypothyroidism | rs2509056 | G | T | -0.03793 | 0.008095 | 2.78E-06 |
| Hypothyroidism | rs2553614 | T | G | -0.0569 | 0.007842 | 4.00E-13 |
| Hypothyroidism | rs2629649 | G | T | 0.050726 | 0.008388 | 1.47E-09 |
| Hypothyroidism | rs268134 | G | A | -0.04 | 0.00833 | 1.58E-06 |
| Hypothyroidism | rs2774290 | T | C | 0.06261 | 0.008299 | 4.55E-14 |
| Hypothyroidism | rs28391281 | C | T | -0.05034 | 0.007834 | 1.31E-10 |
| Hypothyroidism | rs28670946 | G | A | -0.04508 | 0.009102 | 7.32E-07 |
| Hypothyroidism | rs2893970 | C | T | -0.0687 | 0.010547 | 7.34E-11 |
| Hypothyroidism | rs2928167 | G | A | -0.1 | 0.009932 | 7.63E-24 |
| Hypothyroidism | rs2950387 | C | T | 0.056926 | 0.008398 | 1.21E-11 |
| Hypothyroidism | rs2967683 | C | T | -0.04454 | 0.008944 | 6.36E-07 |
| Hypothyroidism | rs2972166 | A | G | -0.03906 | 0.008376 | 3.11E-06 |
| Hypothyroidism | rs2976908 | G | T | -0.06053 | 0.007891 | 1.72E-14 |
| Hypothyroidism | rs2985693 | T | C | -0.05078 | 0.010628 | 1.77E-06 |
| Hypothyroidism | rs3010275 | G | T | 0.054123 | 0.009153 | 3.36E-09 |
| Hypothyroidism | rs30233 | A | G | -0.05403 | 0.007948 | 1.07E-11 |
| Hypothyroidism | rs3103991 | G | A | 0.055828 | 0.009073 | 7.61E-10 |
| Hypothyroidism | rs3130186 | T | C | -0.12903 | 0.009936 | 1.47E-38 |
| Hypothyroidism | rs329118 | T | C | -0.03682 | 0.007932 | 3.46E-06 |
| Hypothyroidism | rs332683 | C | T | 0.03807 | 0.007987 | 1.88E-06 |
| Hypothyroidism | rs333151 | A | G | -0.04544 | 0.008828 | 2.64E-07 |
| Hypothyroidism | rs335640 | C | T | -0.05807 | 0.008062 | 5.93E-13 |
| Hypothyroidism | rs34582796 | G | A | -0.04932 | 0.010624 | 3.44E-06 |
| Hypothyroidism | rs346835 | T | C | -0.04167 | 0.008237 | 4.22E-07 |
| Hypothyroidism | rs34801483 | G | A | 0.065349 | 0.010497 | 4.79E-10 |
| Hypothyroidism | rs35092607 | C | T | -0.09299 | 0.019173 | 1.24E-06 |
| Hypothyroidism | rs35181240 | T | C | -0.06837 | 0.014465 | 2.29E-06 |
| Hypothyroidism | rs35277385 | T | C | 0.037226 | 0.00789 | 2.38E-06 |
| Hypothyroidism | rs35682190 | G | A | -0.05901 | 0.012021 | 9.15E-07 |
| Hypothyroidism | rs35717611 | T | C | 0.053807 | 0.008089 | 2.89E-11 |
| Hypothyroidism | rs35937663 | A | C | -0.14015 | 0.025641 | 4.61E-08 |
| Hypothyroidism | rs36144291 | C | T | -0.0454 | 0.009584 | 2.17E-06 |
| Hypothyroidism | rs3737136 | G | A | -0.05087 | 0.008945 | 1.29E-08 |
| Hypothyroidism | rs3739280 | T | G | 0.342524 | 0.069662 | 8.79E-07 |
| Hypothyroidism | rs377380 | G | A | -0.04755 | 0.009587 | 7.06E-07 |
| Hypothyroidism | rs3778752 | T | G | 0.045252 | 0.007874 | 9.08E-09 |
| Hypothyroidism | rs3780446 | T | C | 0.050373 | 0.008888 | 1.45E-08 |
| Hypothyroidism | rs3781661 | T | C | 0.048657 | 0.010043 | 1.27E-06 |
| Hypothyroidism | rs3788542 | T | C | 0.087677 | 0.017163 | 3.25E-07 |
| Hypothyroidism | rs3814752 | A | G | 0.052395 | 0.010816 | 1.27E-06 |
| Hypothyroidism | rs385251 | A | G | 0.071875 | 0.008102 | 7.23E-19 |
| Hypothyroidism | rs3867608 | G | A | 0.10179 | 0.01983 | 2.85E-07 |
| Hypothyroidism | rs3946137 | G | A | 0.046064 | 0.008188 | 1.85E-08 |
| Hypothyroidism | rs41294627 | G | A | -0.06913 | 0.012244 | 1.64E-08 |
| Hypothyroidism | rs414755 | A | G | 0.051931 | 0.007909 | 5.17E-11 |
| Hypothyroidism | rs4429345 | G | A | -0.03746 | 0.007881 | 2.00E-06 |
| Hypothyroidism | rs453979 | T | C | 0.037478 | 0.008189 | 4.72E-06 |
| Hypothyroidism | rs4606850 | T | C | 0.064999 | 0.010459 | 5.14E-10 |
| Hypothyroidism | rs4675423 | C | T | -0.0387 | 0.007965 | 1.18E-06 |
| Hypothyroidism | rs470113 | G | A | -0.05736 | 0.009594 | 2.25E-09 |
| Hypothyroidism | rs4713874 | C | T | 0.193527 | 0.03386 | 1.09E-08 |
| Hypothyroidism | rs472678 | A | G | -0.0414 | 0.00874 | 2.17E-06 |
| Hypothyroidism | rs4733838 | A | G | -0.04092 | 0.00812 | 4.68E-07 |
| Hypothyroidism | rs4743597 | C | T | 0.0411 | 0.008442 | 1.12E-06 |
| Hypothyroidism | rs4749809 | C | A | -0.06481 | 0.011791 | 3.87E-08 |
| Hypothyroidism | rs4836979 | C | A | -0.08179 | 0.016552 | 7.75E-07 |
| Hypothyroidism | rs4843875 | T | C | 0.048545 | 0.010034 | 1.31E-06 |
| Hypothyroidism | rs484479 | G | A | -0.04308 | 0.008928 | 1.39E-06 |
| Hypothyroidism | rs4853459 | C | T | -0.12348 | 0.00916 | 2.03E-41 |
| Hypothyroidism | rs4933466 | G | A | -0.04307 | 0.007988 | 6.99E-08 |
| Hypothyroidism | rs4936413 | C | T | 0.040134 | 0.007999 | 5.24E-07 |
| Hypothyroidism | rs4988784 | T | C | 0.046276 | 0.009169 | 4.49E-07 |
| Hypothyroidism | rs511973 | A | C | 0.065866 | 0.010194 | 1.04E-10 |
| Hypothyroidism | rs523582 | T | C | 0.040234 | 0.007902 | 3.55E-07 |
| Hypothyroidism | rs55645855 | T | G | 0.062733 | 0.01236 | 3.87E-07 |
| Hypothyroidism | rs55783116 | C | A | 0.044259 | 0.009613 | 4.14E-06 |
| Hypothyroidism | rs56011703 | T | C | 0.0841 | 0.014172 | 2.95E-09 |
| Hypothyroidism | rs56159866 | T | C | -0.05913 | 0.009353 | 2.58E-10 |
| Hypothyroidism | rs56175143 | A | G | 0.226089 | 0.01758 | 7.51E-38 |
| Hypothyroidism | rs56197325 | G | A | -0.04097 | 0.008858 | 3.75E-06 |
| Hypothyroidism | rs563335682 | G | A | -0.09217 | 0.012022 | 1.76E-14 |
| Hypothyroidism | rs56340658 | C | T | 0.076912 | 0.01402 | 4.11E-08 |
| Hypothyroidism | rs57631609 | C | T | 0.05273 | 0.009522 | 3.07E-08 |
| Hypothyroidism | rs57652885 | T | C | -0.10708 | 0.017891 | 2.16E-09 |
| Hypothyroidism | rs576680 | A | C | 0.048517 | 0.007989 | 1.25E-09 |
| Hypothyroidism | rs581694 | C | T | -0.04499 | 0.008647 | 1.95E-07 |
| Hypothyroidism | rs58688157 | G | A | -0.05247 | 0.009752 | 7.42E-08 |
| Hypothyroidism | rs59262563 | A | G | 0.104594 | 0.021305 | 9.14E-07 |
| Hypothyroidism | rs59576148 | G | A | 0.050779 | 0.010393 | 1.03E-06 |
| Hypothyroidism | rs6006220 | T | C | 0.046417 | 0.00891 | 1.90E-07 |
| Hypothyroidism | rs60373740 | G | A | 0.099575 | 0.021721 | 4.55E-06 |
| Hypothyroidism | rs60394424 | A | G | 0.069252 | 0.014235 | 1.14E-06 |
| Hypothyroidism | rs61201527 | C | A | 0.064136 | 0.011765 | 5.00E-08 |
| Hypothyroidism | rs61759532 | T | C | 0.077846 | 0.009924 | 4.37E-15 |
| Hypothyroidism | rs61916675 | G | A | 0.054336 | 0.00839 | 9.43E-11 |
| Hypothyroidism | rs61938844 | A | G | 0.190657 | 0.040668 | 2.76E-06 |
| Hypothyroidism | rs61938962 | T | C | 0.080565 | 0.008332 | 4.09E-22 |
| Hypothyroidism | rs61944361 | G | A | -0.08542 | 0.015768 | 6.06E-08 |
| Hypothyroidism | rs62131895 | C | T | 0.048081 | 0.010361 | 3.48E-06 |
| Hypothyroidism | rs62181736 | C | T | -0.04305 | 0.008334 | 2.41E-07 |
| Hypothyroidism | rs62395778 | A | G | 0.316143 | 0.062462 | 4.16E-07 |
| Hypothyroidism | rs62476229 | G | A | 0.04339 | 0.008531 | 3.65E-07 |
| Hypothyroidism | rs62621812 | A | G | 0.099056 | 0.019133 | 2.25E-07 |
| Hypothyroidism | rs627947 | A | G | -0.03684 | 0.007917 | 3.27E-06 |
| Hypothyroidism | rs6427844 | G | A | 0.039953 | 0.008002 | 5.96E-07 |
| Hypothyroidism | rs6456465 | A | G | 0.060081 | 0.011097 | 6.15E-08 |
| Hypothyroidism | rs6471875 | A | C | 0.048991 | 0.007971 | 7.93E-10 |
| Hypothyroidism | rs6511611 | T | C | 0.040462 | 0.008829 | 4.59E-06 |
| Hypothyroidism | rs6562772 | T | C | -0.04021 | 0.007813 | 2.65E-07 |
| Hypothyroidism | rs661891 | C | A | -0.04342 | 0.007833 | 2.97E-08 |
| Hypothyroidism | rs6672745 | T | C | 0.074338 | 0.015542 | 1.73E-06 |
| Hypothyroidism | rs66760320 | T | C | -0.06379 | 0.009803 | 7.64E-11 |
| Hypothyroidism | rs6678885 | C | A | 0.054504 | 0.010101 | 6.81E-08 |
| Hypothyroidism | rs6690837 | C | T | 0.062936 | 0.009899 | 2.05E-10 |
| Hypothyroidism | rs6720896 | C | T | 0.042471 | 0.00896 | 2.13E-06 |
| Hypothyroidism | rs6724073 | C | T | -0.08061 | 0.008081 | 1.96E-23 |
| Hypothyroidism | rs6724363 | T | G | 0.089522 | 0.008107 | 2.38E-28 |
| Hypothyroidism | rs6727170 | C | T | 0.047144 | 0.009316 | 4.18E-07 |
| Hypothyroidism | rs6729311 | G | A | -0.08308 | 0.014629 | 1.36E-08 |
| Hypothyroidism | rs67420220 | G | A | 0.091515 | 0.018154 | 4.63E-07 |
| Hypothyroidism | rs67514601 | G | A | 0.042722 | 0.008271 | 2.40E-07 |
| Hypothyroidism | rs679704 | A | G | 0.038445 | 0.008176 | 2.58E-06 |
| Hypothyroidism | rs686752 | G | A | -0.04728 | 0.009049 | 1.74E-07 |
| Hypothyroidism | rs6895555 | A | G | 0.036182 | 0.007879 | 4.38E-06 |
| Hypothyroidism | rs6901423 | G | A | 0.040377 | 0.008027 | 4.90E-07 |
| Hypothyroidism | rs6902545 | A | G | 0.044338 | 0.008179 | 5.93E-08 |
| Hypothyroidism | rs6920220 | A | G | 0.048859 | 0.009889 | 7.79E-07 |
| Hypothyroidism | rs6934244 | A | C | 0.083375 | 0.016954 | 8.75E-07 |
| Hypothyroidism | rs6994357 | C | T | 0.057897 | 0.0118 | 9.27E-07 |
| Hypothyroidism | rs7004509 | G | T | -0.04234 | 0.007876 | 7.61E-08 |
| Hypothyroidism | rs7097397 | A | G | -0.04019 | 0.008147 | 8.07E-07 |
| Hypothyroidism | rs7132512 | A | C | -0.04074 | 0.007961 | 3.10E-07 |
| Hypothyroidism | rs713427 | C | T | 0.057414 | 0.009369 | 8.88E-10 |
| Hypothyroidism | rs7138373 | G | T | 0.044897 | 0.008674 | 2.27E-07 |
| Hypothyroidism | rs71430783 | T | G | 0.09297 | 0.01006 | 2.43E-20 |
| Hypothyroidism | rs7162561 | C | T | 0.047418 | 0.008837 | 8.07E-08 |
| Hypothyroidism | rs71641308 | T | C | 0.074595 | 0.013166 | 1.46E-08 |
| Hypothyroidism | rs7169981 | A | C | -0.04002 | 0.008402 | 1.91E-06 |
| Hypothyroidism | rs7195507 | G | A | -0.04131 | 0.008463 | 1.05E-06 |
| Hypothyroidism | rs724078 | A | G | -0.05211 | 0.007947 | 5.47E-11 |
| Hypothyroidism | rs72729322 | T | C | 0.057454 | 0.009559 | 1.85E-09 |
| Hypothyroidism | rs72763208 | A | C | -0.07876 | 0.016327 | 1.41E-06 |
| Hypothyroidism | rs72796365 | T | C | 0.141199 | 0.023939 | 3.67E-09 |
| Hypothyroidism | rs7309495 | T | C | 0.064808 | 0.012795 | 4.08E-07 |
| Hypothyroidism | rs73245731 | G | T | 0.056769 | 0.011829 | 1.59E-06 |
| Hypothyroidism | rs73290707 | T | C | 0.111264 | 0.023357 | 1.90E-06 |
| Hypothyroidism | rs735000 | T | C | 0.076572 | 0.011903 | 1.25E-10 |
| Hypothyroidism | rs7350442 | T | C | 0.041532 | 0.008673 | 1.68E-06 |
| Hypothyroidism | rs74351530 | A | G | 0.160258 | 0.028576 | 2.05E-08 |
| Hypothyroidism | rs74531406 | A | G | 0.056423 | 0.011898 | 2.12E-06 |
| Hypothyroidism | rs7454888 | T | C | 0.094218 | 0.016621 | 1.44E-08 |
| Hypothyroidism | rs74725952 | T | C | -0.08817 | 0.014977 | 3.92E-09 |
| Hypothyroidism | rs74745605 | G | A | 0.078498 | 0.009224 | 1.74E-17 |
| Hypothyroidism | rs75054873 | C | T | -0.15958 | 0.033832 | 2.40E-06 |
| Hypothyroidism | rs75098738 | A | G | 0.196763 | 0.041167 | 1.76E-06 |
| Hypothyroidism | rs75125154 | G | A | 0.206319 | 0.011894 | 2.07E-67 |
| Hypothyroidism | rs75315114 | G | A | 0.133749 | 0.028837 | 3.52E-06 |
| Hypothyroidism | rs75329808 | A | G | -0.09384 | 0.013165 | 1.02E-12 |
| Hypothyroidism | rs7536032 | G | A | -0.04277 | 0.007956 | 7.63E-08 |
| Hypothyroidism | rs7559184 | G | T | 0.134449 | 0.026857 | 5.55E-07 |
| Hypothyroidism | rs759049 | A | G | -0.03921 | 0.007908 | 7.09E-07 |
| Hypothyroidism | rs7604427 | G | A | -0.0426 | 0.008507 | 5.51E-07 |
| Hypothyroidism | rs7608632 | G | T | -0.0459 | 0.008131 | 1.65E-08 |
| Hypothyroidism | rs76169968 | A | G | -0.08611 | 0.013355 | 1.14E-10 |
| Hypothyroidism | rs76307396 | A | G | 0.078288 | 0.016183 | 1.31E-06 |
| Hypothyroidism | rs76392636 | G | A | 0.114047 | 0.022682 | 4.96E-07 |
| Hypothyroidism | rs76428106 | C | T | 0.24652 | 0.034409 | 7.81E-13 |
| Hypothyroidism | rs76631016 | T | C | 0.104774 | 0.021282 | 8.51E-07 |
| Hypothyroidism | rs76653830 | T | C | 0.06331 | 0.012777 | 7.23E-07 |
| Hypothyroidism | rs76737444 | C | T | 0.118816 | 0.022693 | 1.64E-07 |
| Hypothyroidism | rs76796396 | T | C | 0.091911 | 0.018302 | 5.11E-07 |
| Hypothyroidism | rs76896383 | G | A | 0.103064 | 0.022152 | 3.28E-06 |
| Hypothyroidism | rs7701443 | G | A | -0.04686 | 0.008133 | 8.30E-09 |
| Hypothyroidism | rs77184138 | T | C | -0.04344 | 0.009304 | 3.02E-06 |
| Hypothyroidism | rs77201110 | C | T | -0.05463 | 0.010812 | 4.34E-07 |
| Hypothyroidism | rs7734027 | A | G | 0.052415 | 0.008644 | 1.33E-09 |
| Hypothyroidism | rs7738250 | A | G | -0.11592 | 0.013165 | 1.30E-18 |
| Hypothyroidism | rs774121 | C | T | -0.07116 | 0.008971 | 2.16E-15 |
| Hypothyroidism | rs77916927 | G | A | -0.1135 | 0.020536 | 3.25E-08 |
| Hypothyroidism | rs78067210 | T | C | -0.09705 | 0.020731 | 2.85E-06 |
| Hypothyroidism | rs78199107 | C | T | -0.09857 | 0.018982 | 2.07E-07 |
| Hypothyroidism | rs78257559 | G | A | 0.078348 | 0.016635 | 2.48E-06 |
| Hypothyroidism | rs78492684 | G | A | 0.056077 | 0.011802 | 2.02E-06 |
| Hypothyroidism | rs78783493 | T | C | -0.05471 | 0.009406 | 6.00E-09 |
| Hypothyroidism | rs78953577 | T | G | -0.06138 | 0.009118 | 1.67E-11 |
| Hypothyroidism | rs7901053 | A | G | 0.037033 | 0.008068 | 4.43E-06 |
| Hypothyroidism | rs7902146 | T | C | -0.0732 | 0.008984 | 3.71E-16 |
| Hypothyroidism | rs79259951 | C | A | -0.08133 | 0.016043 | 3.98E-07 |
| Hypothyroidism | rs793094 | A | G | -0.04019 | 0.007852 | 3.08E-07 |
| Hypothyroidism | rs79478518 | C | T | 0.121739 | 0.023984 | 3.86E-07 |
| Hypothyroidism | rs794999 | G | A | -0.06796 | 0.009258 | 2.13E-13 |
| Hypothyroidism | rs80021475 | A | G | -0.09509 | 0.020811 | 4.89E-06 |
| Hypothyroidism | rs8006310 | G | A | -0.0543 | 0.007832 | 4.12E-12 |
| Hypothyroidism | rs8086051 | T | C | 0.062668 | 0.010843 | 7.49E-09 |
| Hypothyroidism | rs8193 | T | C | 0.058247 | 0.008184 | 1.10E-12 |
| Hypothyroidism | rs836487 | T | C | 0.037441 | 0.007834 | 1.76E-06 |
| Hypothyroidism | rs853884 | G | A | 0.071219 | 0.014158 | 4.90E-07 |
| Hypothyroidism | rs888157 | G | A | -0.03858 | 0.008343 | 3.76E-06 |
| Hypothyroidism | rs893823 | G | T | 0.037054 | 0.007968 | 3.31E-06 |
| Hypothyroidism | rs9267873 | T | C | 0.064762 | 0.007796 | 9.77E-17 |
| Hypothyroidism | rs927985 | A | G | -0.07565 | 0.011375 | 2.91E-11 |
| Hypothyroidism | rs9292 | G | A | -0.12431 | 0.021927 | 1.43E-08 |
| Hypothyroidism | rs9350354 | G | A | 0.037298 | 0.008055 | 3.65E-06 |
| Hypothyroidism | rs9368744 | T | C | 0.179345 | 0.022871 | 4.45E-15 |
| Hypothyroidism | rs9378805 | C | A | 0.045409 | 0.007838 | 6.91E-09 |
| Hypothyroidism | rs938726 | C | A | -0.06761 | 0.008767 | 1.24E-14 |
| Hypothyroidism | rs9389300 | A | G | -0.03708 | 0.008109 | 4.83E-06 |
| Hypothyroidism | rs9393698 | A | G | -0.04963 | 0.007958 | 4.48E-10 |
| Hypothyroidism | rs9497965 | T | C | 0.065586 | 0.008276 | 2.29E-15 |
| Hypothyroidism | rs9562953 | G | A | 0.070128 | 0.013851 | 4.12E-07 |
| Hypothyroidism | rs9784818 | A | G | -0.04728 | 0.009721 | 1.15E-06 |
| Hypothyroidism | rs9794362 | T | C | 0.043858 | 0.008887 | 8.01E-07 |
| Hypothyroidism | rs9847942 | A | G | 0.04795 | 0.010426 | 4.25E-06 |
| Hypothyroidism | rs9859406 | A | G | -0.04294 | 0.00855 | 5.11E-07 |
| Hypothyroidism | rs9880421 | T | C | -0.04128 | 0.00832 | 6.99E-07 |
| Hypothyroidism | rs9923850 | C | A | 0.039382 | 0.008147 | 1.34E-06 |
| Hypothyroidism | rs9981704 | T | C | 0.056132 | 0.010143 | 3.13E-08 |
| Hyperthyroidism | rs10168266 | T | C | 0.186518 | 0.040151 | 3.39E-06 |
| Hyperthyroidism | rs10947129 | A | G | -0.17795 | 0.038797 | 4.50E-06 |
| Hyperthyroidism | rs11008857 | C | T | 0.186014 | 0.0389 | 1.74E-06 |
| Hyperthyroidism | rs11150188 | G | A | 0.195269 | 0.035137 | 2.74E-08 |
| Hyperthyroidism | rs115166974 | T | C | 0.426022 | 0.091251 | 3.03E-06 |
| Hyperthyroidism | rs11575073 | T | C | 0.259437 | 0.056782 | 4.90E-06 |
| Hyperthyroidism | rs11754183 | A | G | -0.38817 | 0.078936 | 8.77E-07 |
| Hyperthyroidism | rs1195263 | A | G | 0.350405 | 0.067919 | 2.48E-07 |
| Hyperthyroidism | rs12189725 | T | G | -0.24551 | 0.051395 | 1.78E-06 |
| Hyperthyroidism | rs140239646 | A | G | 0.351897 | 0.076577 | 4.32E-06 |
| Hyperthyroidism | rs147916535 | A | C | -0.29023 | 0.060965 | 1.93E-06 |
| Hyperthyroidism | rs182700050 | T | C | 0.540666 | 0.063807 | 2.38E-17 |
| Hyperthyroidism | rs1907690 | T | C | 0.161039 | 0.034793 | 3.68E-06 |
| Hyperthyroidism | rs191757729 | A | G | -1.09348 | 0.23812 | 4.39E-06 |
| Hyperthyroidism | rs236448 | C | A | 0.168725 | 0.035172 | 1.61E-06 |
| Hyperthyroidism | rs2516455 | T | G | 0.262991 | 0.036758 | 8.39E-13 |
| Hyperthyroidism | rs2524106 | A | G | -0.25261 | 0.047402 | 9.87E-08 |
| Hyperthyroidism | rs28366263 | C | T | -0.36159 | 0.063085 | 9.94E-09 |
| Hyperthyroidism | rs3988342 | A | C | 0.172773 | 0.034105 | 4.07E-07 |
| Hyperthyroidism | rs405011 | T | C | 0.161541 | 0.034606 | 3.04E-06 |
| Hyperthyroidism | rs4576262 | A | C | 0.181619 | 0.039207 | 3.62E-06 |
| Hyperthyroidism | rs6072797 | G | A | 0.23117 | 0.043774 | 1.28E-07 |
| Hyperthyroidism | rs6074022 | T | C | 0.212511 | 0.038344 | 2.99E-08 |
| Hyperthyroidism | rs60946162 | T | C | -0.16933 | 0.03391 | 5.93E-07 |
| Hyperthyroidism | rs62031985 | T | C | 0.384081 | 0.081484 | 2.43E-06 |
| Hyperthyroidism | rs6832151 | T | G | -0.18133 | 0.037221 | 1.11E-06 |
| Hyperthyroidism | rs72891915 | A | G | 0.64032 | 0.069909 | 5.22E-20 |
| Hyperthyroidism | rs75127309 | G | A | 0.316558 | 0.065295 | 1.25E-06 |
| Hyperthyroidism | rs75760731 | A | C | 0.539777 | 0.086805 | 5.03E-10 |
| Hyperthyroidism | rs7749579 | T | C | 0.303973 | 0.057435 | 1.21E-07 |
| Hyperthyroidism | rs7769527 | G | A | 0.388148 | 0.056357 | 5.68E-12 |
| Hyperthyroidism | rs80054410 | C | T | 0.204922 | 0.035685 | 9.33E-09 |
| Hyperthyroidism | rs942495 | T | C | 0.382603 | 0.057451 | 2.75E-11 |
| Graves' disease | rs10199135 | G | A | -0.19316 | 0.039828 | 1.23E-06 |
| Graves' disease | rs10995105 | C | T | 0.156743 | 0.032496 | 1.41E-06 |
| Graves' disease | rs112796164 | G | T | 0.445244 | 0.037469 | 1.45E-32 |
| Graves' disease | rs117366411 | T | C | 0.94392 | 0.206169 | 4.69E-06 |
| Graves' disease | rs12482947 | C | T | 0.173963 | 0.027244 | 1.71E-10 |
| Graves' disease | rs13003541 | G | A | 0.557909 | 0.107706 | 2.22E-07 |
| Graves' disease | rs138242238 | T | C | -0.26762 | 0.048737 | 3.99E-08 |
| Graves' disease | rs140103824 | G | T | -0.53458 | 0.082029 | 7.17E-11 |
| Graves' disease | rs140430242 | T | C | 0.39962 | 0.078681 | 3.79E-07 |
| Graves' disease | rs149465829 | T | C | 0.356041 | 0.052922 | 1.72E-11 |
| Graves' disease | rs1565355 | T | C | 0.261131 | 0.045233 | 7.79E-09 |
| Graves' disease | rs1569723 | A | C | 0.182338 | 0.030771 | 3.11E-09 |
| Graves' disease | rs16833241 | C | T | -0.12718 | 0.026705 | 1.91E-06 |
| Graves' disease | rs17513810 | C | A | -0.44281 | 0.085444 | 2.19E-07 |
| Graves' disease | rs1997397 | C | T | 0.127441 | 0.027672 | 4.12E-06 |
| Graves' disease | rs2048496 | G | A | -0.17927 | 0.03881 | 3.85E-06 |
| Graves' disease | rs2160215 | C | T | 0.320977 | 0.027796 | 7.58E-31 |
| Graves' disease | rs2269134 | C | T | 0.142759 | 0.029264 | 1.07E-06 |
| Graves' disease | rs2395568 | G | A | -0.37052 | 0.071673 | 2.35E-07 |
| Graves' disease | rs2395617 | C | A | 0.259985 | 0.05134 | 4.11E-07 |
| Graves' disease | rs2792043 | C | A | 0.14022 | 0.026587 | 1.34E-07 |
| Graves' disease | rs34953613 | C | T | -0.25539 | 0.055233 | 3.77E-06 |
| Graves' disease | rs35811474 | T | C | -0.19197 | 0.039794 | 1.41E-06 |
| Graves' disease | rs4338740 | C | T | 0.148498 | 0.028232 | 1.44E-07 |
| Graves' disease | rs498114 | T | G | 0.227957 | 0.031975 | 1.01E-12 |
| Graves' disease | rs60946162 | T | C | -0.1613 | 0.027366 | 3.77E-09 |
| Graves' disease | rs6667564 | C | T | -0.13103 | 0.027985 | 2.84E-06 |
| Graves' disease | rs6943937 | G | A | -0.13329 | 0.028364 | 2.61E-06 |
| Graves' disease | rs6990534 | G | A | 0.133851 | 0.028792 | 3.34E-06 |
| Graves' disease | rs72928038 | A | G | 0.192593 | 0.039634 | 1.18E-06 |
| Graves' disease | rs7738118 | A | G | -0.23139 | 0.035217 | 5.02E-11 |
| Graves' disease | rs7749579 | T | C | 0.215868 | 0.046943 | 4.25E-06 |
| Graves' disease | rs77746120 | G | T | 0.476406 | 0.09546 | 6.02E-07 |
| Graves' disease | rs80217563 | T | C | 0.232794 | 0.050122 | 3.41E-06 |
| Graves' disease | rs9263773 | C | T | 0.150435 | 0.026853 | 2.12E-08 |
| Hashimoto thyroiditis | rs1025256 | G | A | 0.314269 | 0.065123 | 1.39E-06 |
| Hashimoto thyroiditis | rs3116997 | A | G | -0.48694 | 0.090937 | 8.57E-08 |
| Hashimoto thyroiditis | rs6679677 | A | C | 0.561915 | 0.077893 | 5.44E-13 |
| Hashimoto thyroiditis | rs9271365 | G | T | 0.457431 | 0.061198 | 7.74E-14 |

**
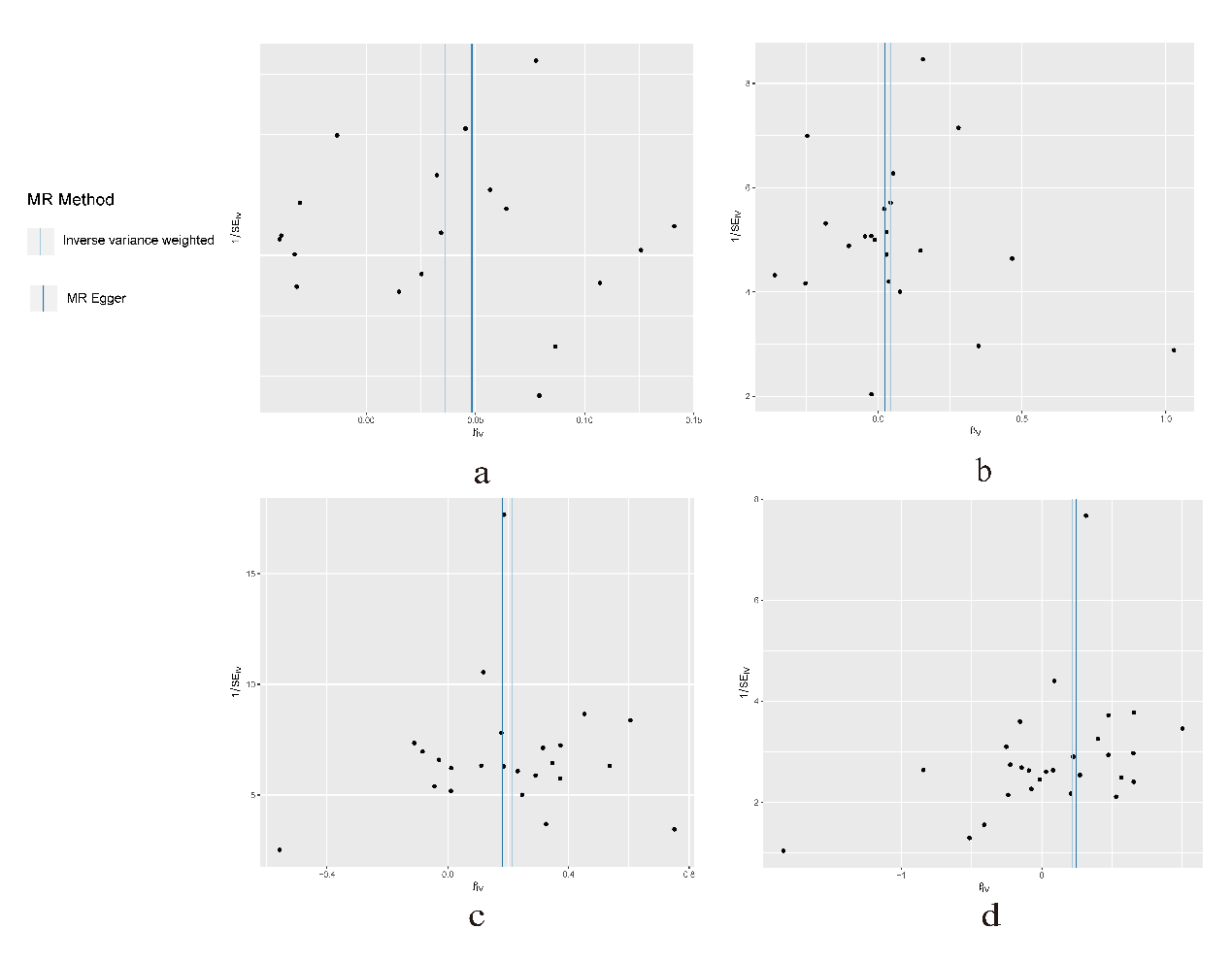
**

**Supplementary Fig. 1** Funnel plots of the causal effect of MG on AITD. (a) Funnel plot of the causal effect of MG on autoimmune hypothyroidism; (b) Funnel plot of the causal effect of MG on Autoimmune hyperthyroidism; (c) Funnel plot of the causal effect of MG on Graves' disease; (d) Funnel plot of the causal effect of MG on Hashimoto's thyroiditis.

**
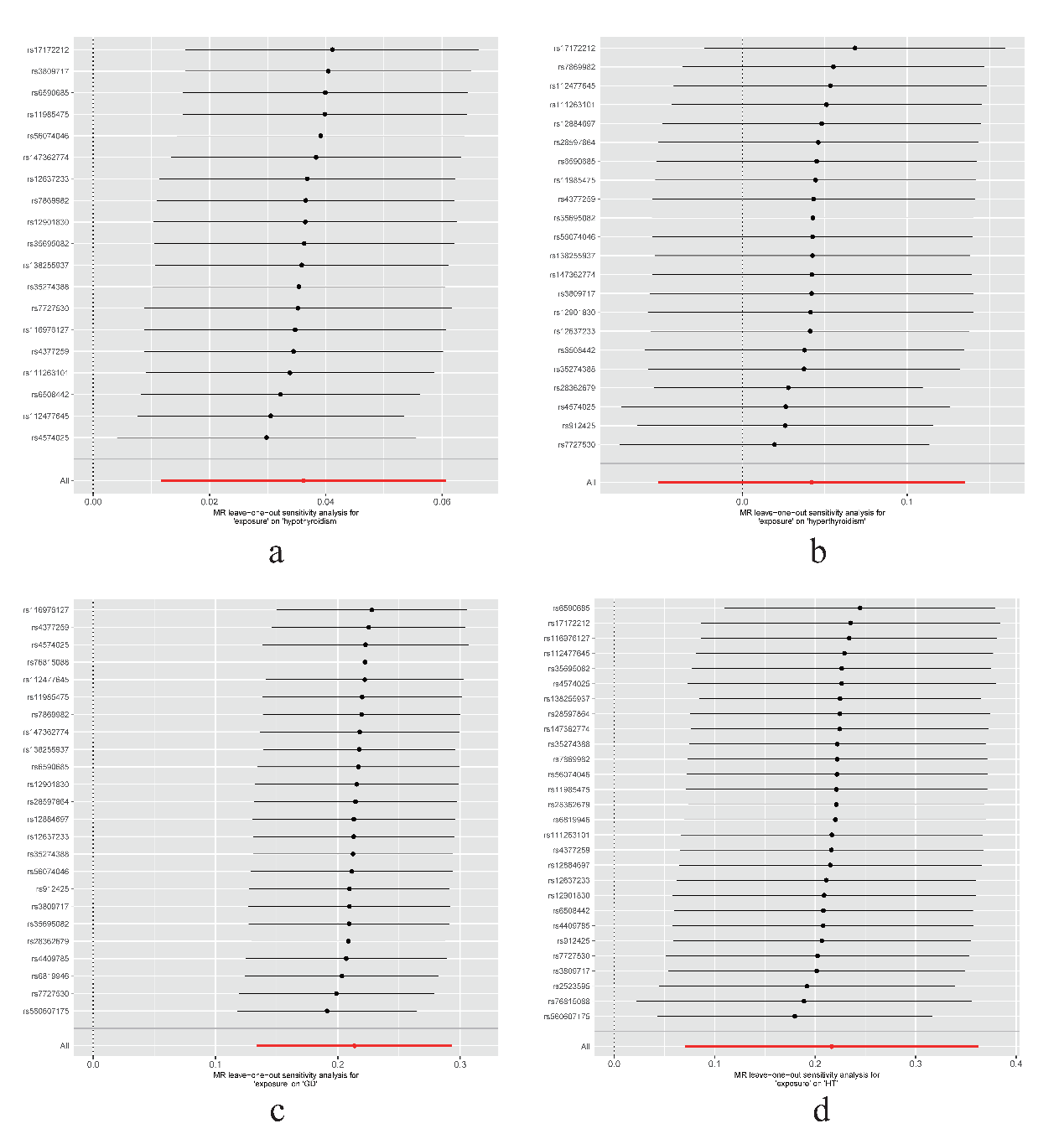
**

**Supplementary Fig. 2**  Leave-one-out analysis of the causal effect of MG on AITD. (a) Leave-one-out analysis of the causal effect of MG on autoimmune hypothyroidism; (b) Leave-one-out analysis of the causal effect of MG on Autoimmune hyperthyroidism; (c) Leave-one-out analysis of the causal effect of MG on Graves' disease; (d) Leave-one-out analysis of the causal effect of MG on Hashimoto's thyroiditis.


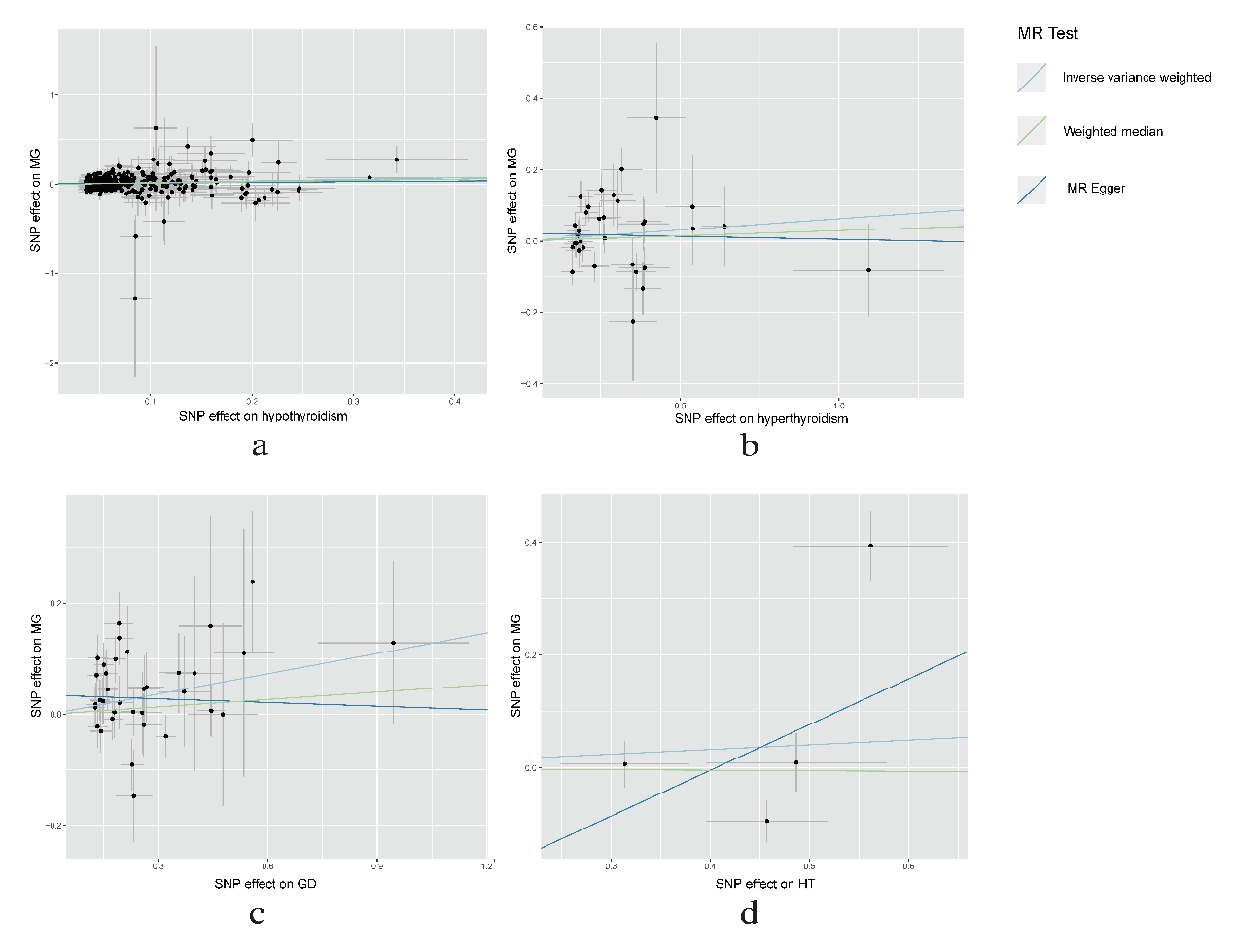


**Supplementary Fig. 3** Scatter plots of the causal effect of AITD on MG. (a) Scatter plot of the causal effect of autoimmune hypothyroidism on MG; (b) Scatter plot of the causal effect of autoimmune hyperthyroidism on MG; (c) Scatter plot of the causal effect of Graves' disease on MG; (d) Scatter plot of the causal effect of Hashimoto's thyroiditis on MG.


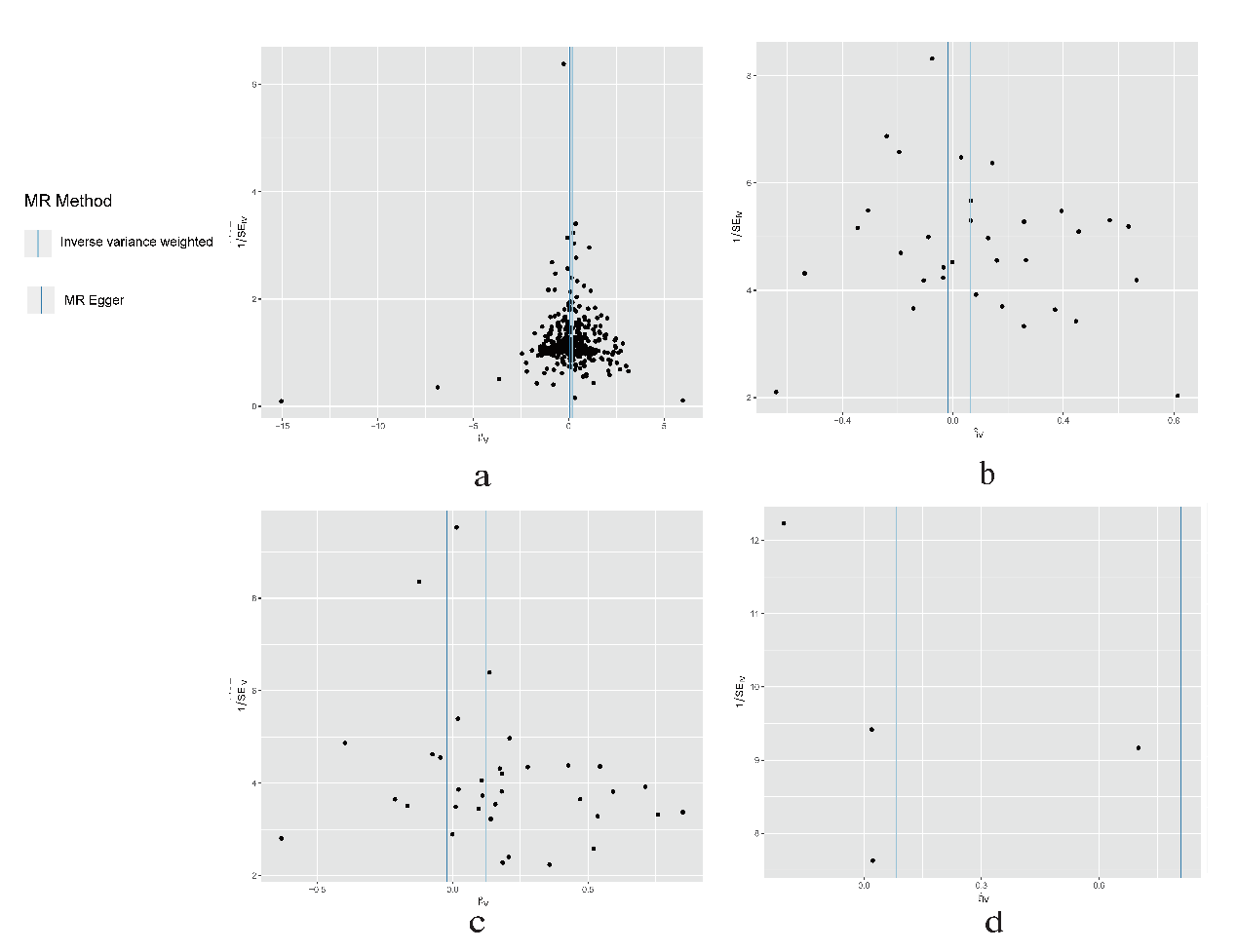


**Supplementary Fig. 4** Funnel plots of the causal effect of AITD on MG. (a) Funnel plot of the causal effect of autoimmune hypothyroidism on MG; (b) Funnel plot of the causal effect of autoimmune hyperthyroidism on MG; (c) Funnel plot of the causal effect of Graves' disease on MG; (d) Funnel plot of the causal effect of Hashimoto's thyroiditis on MG.


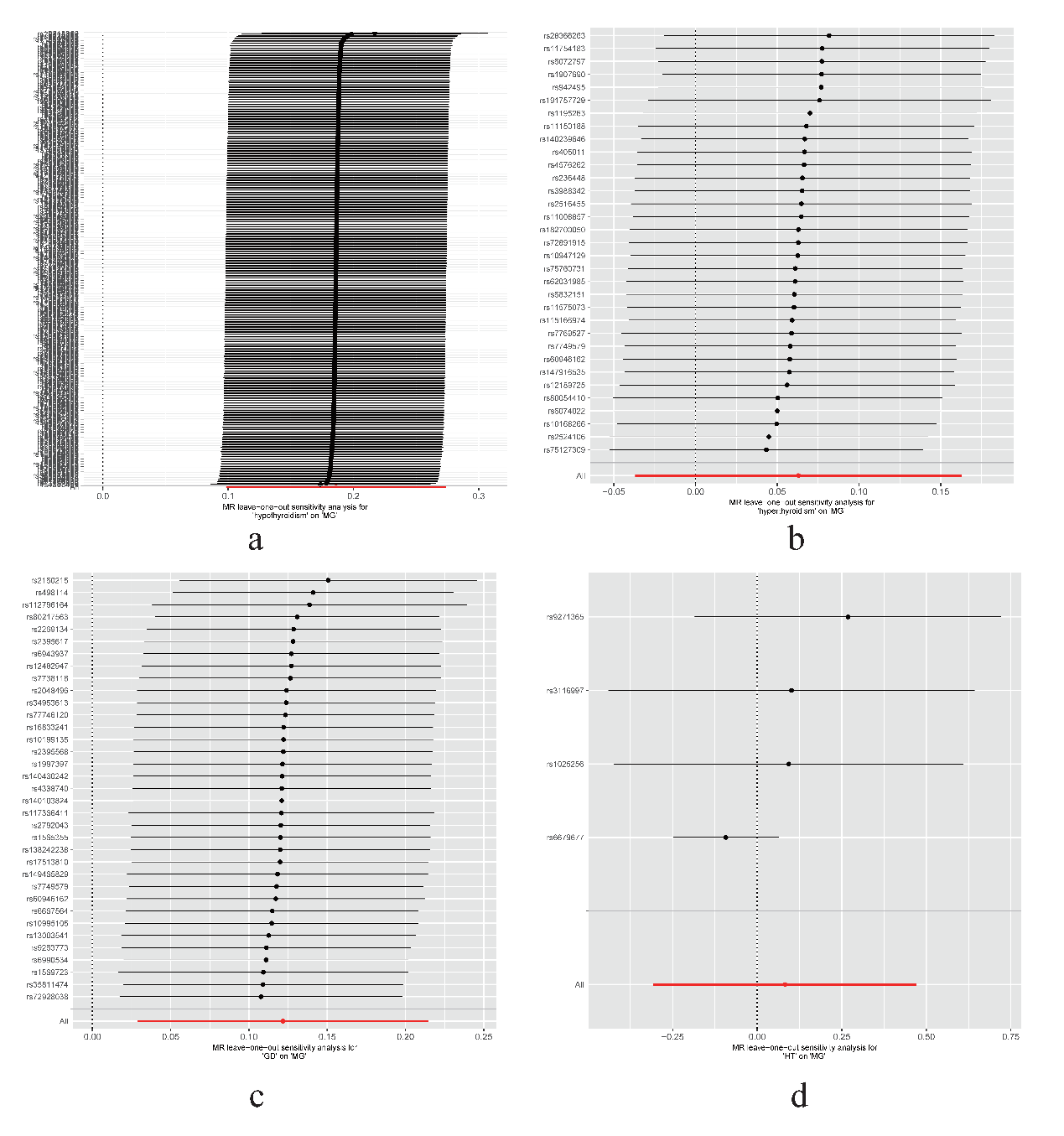


**Supplementary Fig. 5**  Leave-one-out plots of the causal effect of AITD on MG. (a) Leave-one-out plot of the causal effect of autoimmune hypothyroidism on MG; (b) Leave-one-out plot of the causal effect of autoimmune hyperthyroidism on MG; (c) Leave-one-out plot of the causal effect of Graves' disease on MG; (d) Leave-one-out plot of the causal effect of Hashimoto's thyroiditis on MG.
